# Towards maximizing optical access through Smart Dura

**DOI:** 10.1088/1741-2552/ae8eb3

**Published:** 2026-10-06

**Authors:** Sergio Montalvo Vargo, Nari Hong, Gaku Hatanaka, Vishal Jain, Hefu Pan, Ruikang K Wang, Wyeth Bair, Azadeh Yazdan-Shahmorad, Maysamreza Chamanzar

**Affiliations:** 1 Department of Electrical and Computer Engineering, Carnegie Mellon University, Pittsburgh, PA 15213, United States of America; 2 Department of Bioengineering, University of Washington, Seattle, WA 98195, United States of America; 3 Washington National Biomedical Research Center, Seattle, WA, 98195, United States of America; 4 Department of Neurobiology and Biophysics, University of Washington, Seattle, WA 98195, United States of America; 5 Department of Electrical and Computer Engineering, University of Washington, Seattle, WA 98195, United States of America; 6 Department of Biomedical Engineering, Carnegie Mellon University, Pittsburgh, PA 15213, United States of America; 7 Carnegie Mellon Neuroscience Institute, Pittsburgh, PA 15213, United States of America

**Keywords:** transparent *μ*ECoG, Smart Dura, multimodal neural interfaces, multiphoton imaging, intrinsic wide-field imaging, non-human primates, ITO microelectrodes

## Abstract

*Objective.* To overcome the limitations of optical access caused by opaque electrodes in multimodal neural interfaces used in large‐animal models, and to enable scalable, high‐density electrophysiology with unobstructed optical imaging and optogenetic stimulation. *Approach.* In this work, we redesigned our previously demonstrated multimodal cortical surface array, the Smart Dura, by incorporating transparent indium tin oxide (ITO) electrodes. ITO provides good electrical conductivity, while providing high levels of transparency. We discuss the design, microfabrication, benchtop characterization and *in vivo* demonstration of transparent Smart Dura for multimodal brain interfacing. *In vivo* experiments were designed to show the feasibility of simultaneous electrophysiological recording with localized functional optical imaging through transparent Smart Dura in response to visual stimulation. *Main results.* The implementation of ITO electrodes increased the effective optical access of Smart Dura to 100%, eliminating obstruction from traditional metal electrodes. Bench‐top characterization confirmed the electrical and optical performance of the transparent electrodes. *In vivo* results demonstrate direct visual access underneath the ITO electrodes for optical recording and perturbation with co-located simultaneous high-fidelity electrophysiological recording, validating the multimodal capabilities of the transparent Smart Dura. *Significance.* The transparent Smart Dura design removes major barriers to optically integrated, high‐density electrophysiology in large‐animal models and the number and density of ITO electrodes on Smart Dura can be increased without compromising the optical access. This platform advances multimodal neural interfacing in non-human primates and provides unique opportunities to study brain function in health and disease and design therapeutics that can readily translate to humans.

## 1. Introduction

Advances in neural interface technologies have enabled neuroscientists to vastly increase the amount and types of data that can be extracted from neural circuits within the brain based on two main modalities of electrical recording and functional optical imaging [1]. In each of these modalities there are inherent tradeoffs between spatiotemporal resolution and coverage area. For example, within electrical interfaces, penetrating probes provide single- and multi-unit electrophysiological recording with high spatial and temporal resolution of nearby neurons in a small local volume perpendicular to the brain surface [2, 3]. Electrocorticography (ECoG) is commonly used to record local field potential (LFP) activity of extended brain regions on top of the brain surface and provide insights about the underlying network connectivity [2, 3]. Penetrating probes and ECoG arrays can also be used to inject currents into the brain tissue for neural stimulation but cannot target specific cell types and the spatial resolution is limited. In order to address the limitations of electrical modalities, optical methods have been increasingly adopted as complementary techniques to interface with the brain. For example, optical techniques such as hemodynamic wide-field intrinsic signal optical imaging (ISOI) enable functional imaging with high spatial resolution across large continuous regions but have difficulty in resolving fast neuronal dynamics [4]. Fluorescence calcium imaging provides structural and functional imaging of neural spiking activity but over relatively small fields of view [5]. For neural stimulation, optogenetics provides cell-type specific neuromodulation with high spatial and temporal resolution [6] but requires genetic modification. Other imaging modalities such as optical coherence tomography angiography (OCTA) enable imaging blood flow [7, 8] which provide complementary information to electrophysiological recording. This is particularly useful when investigating conditions, such as stroke, that involve neural tissue and vasculature malfunction [9, 10] or when designing neuromodulation therapies to induce neuroplasticity [11]. A multimodal interface that enables simultaneous electrical and optical interfacing with the brain and offers the advantages of these individual functionalities can be a powerful tool for integrating complementary measurements and manipulations with unprecedented spatiotemporal resolution.

We have recently introduced a surface neural interface called Smart Dura, which can replace a section of native dura to provide functional access to the neural tissue of non-human primates (NHPs) for large-scale, high-resolution electrophysiological recording and high optical access. Electro-optical neural interfacing over large areas of NHP brain can bring relevant translational insights to study brain function and dysfunction in humans given their high cognitive and behavioral complexity [12, 13]. Compared to other multimodal technologies recently developed for NHP brain interfacing [14–16], Smart Dura offers higher spatial resolution for neural recording using higher density of its electrodes and very high optical access enabled by its micron-scale metal traces [17–19]. Despite all these advantages, the previous generation of Smart Dura was designed using metals to implement opaque electrodes and traces, limiting its optical access locally under these opaque features. Here, we show that Smart Dura can be designed with transparent electrodes for interfacing with the NHP brain.

To improve the optical access of Smart Dura, a transparent material must be used to replace metallic features of the Smart Dura without compromising electrophysiological recording capabilities. This requires a material that is transparent, conductive and enables electrochemical transduction. Additionally, these materials must be biocompatible as well as compatible with microfabrication. Table 1 summarizes the most relevant materials that offer optical transparency and conductivity. There is no one material that clearly stands out as providing all the desired properties of a transparent and conductive material that can be directly integrated into flexible substrates for interfacing with brain tissue. Given its high transparency, conductivity, and direct compatibility with microfabrication processes, indium tin oxide (ITO) is a good choice. Unfortunately, it is brittle and can crack upon bending and when subjected to flexural forces, as reported in Ledochowitsch *et al* [20], where *µ*ECoG arrays made entirely out of ITO suffered from cracks upon bending, even during handling. Similarly, graphene stands out given its strength and mechanical flexibility as well as its high conductivity. However, challenges of graphene transfer during microfabrication processes [21] limit high yield production of graphene-based devices. Additionally, graphene alone has a low quantum capacitance, resulting in a large electrochemical impedance, compromising the electrochemical transduction of neural signals to electrical signals [21]. Modified PEDOT:PSS thin layer microelectrodes [22, 23] have been made transparent after extensive treatment with acids and other chemicals to improve the conductivity of the native transparent PEDOT:PSS film. However, biocompatibility risks are associated with trace amounts of hazardous acids (i.e. phosphoric acid [23], or dodecyl benzenesulfonic acid [22]) potentially causing adverse effects in biological tissue. Other innovative approaches include using ultra-thin metal-polymer hybrids [24] or ultra-thin nitride materials, i.e. TiN [25], for neural interface electrodes to leverage their conductivity, while providing reasonably high transparency.

**Table 1.** Comparison of different materials used for transparent electrodes for neural interfaces. As it can be analyzed from the table, there is no one material that offers all desired properties for the implementation of transparent electrodes for flexible opto-electrical neural interfaces.

	Indium tin oxide (ITO)	Graphene	Polymer-metal hybrids (8 nm Au + (PIE/PSS)_5_)	Modified PEDOT:PSS
Transparency/Transmittance(%)	**90%** for 100 nm thick films [20]^a^ **55%–80%** for 200 nm thick films [26]	**98%** for 1-layer [27] **87%–92**% for 2-layer w/ HNO_3_ dopant [21] **90%** for 4-layers [28]	**77%** [24]	**∼70%** at 940 nm for 500 nm thick films [22] **70%–75%** for 30 nm thick films [23]

Resistivity (*µ*Ω∙cm)	**480.7** [20]	Not reported	**72.4**[24]	**294** [23]

Sheet Resistance (Ω/□)	**43.7** for 100 nm [20] **12** for 200 nm w/ pre-oxidize step [26]	**125** for 1-layer [27] **276** w/ HNO_3_ [21] **76** for 4-layers [28]	**7.8** [24]	**89** [23]

EIS impedance magnitude at 1 kHz	**3.22 ± 0.48 MΩ** for 20 *µ*m diameter (⌀)^a^**2.52 ± 0.26 MΩ** for ⌀ = 30 *µ*m^a^ **1.72 ± 0.18 MΩ** for ⌀ = 40 *µ*m^a^	**5.4 ± 1.1 MΩ** for ⌀ = 20 *µ*m [21] **243.5 ± 5.9** **kΩ** for ⌀ = 200 *µ*m [28] **541 kΩ** for 50 × 50 *µ*m^2^ [29]	**294 ± 63.4 kΩ** for ⌀ = 30 *µ*m [24]	**45.8 kΩ** for 20 × 20 *µ*m^2^ [23]

Light-induced artifacts	Artifacts reported but can be addressed with high pass filtering	Artifact free [21, 29] Artifacts reported but filtered out because they have a different signature than neural signals [28]	Artifacts reported but can be addressed with high pass filtering. [24]	Artifact free [23]

Flexibility/Mechanical properties	Mechanical properties similar to ceramics, **brittle**	Strong and **flexible**	**Flexible** (bending radius of **1 mm**) [24]	**Flexible** [23]

Fabrication considerations	Deposition of ITO is an established process well documented from semiconductor industry	Single/multi-layer graphene deposition techniques are more novel and less optimized. High fabrication yield is challenging.	Deposition of thin layers of Au is an established process well documented from semiconductor industry	Requires additional non-standard treatments of PEDOT:PSS to improve conductivity, including hazardous acids (i.e. H_3_PO_4_ [23], or dodecyl benzenesulfonic acid [22])

Other considerations		Single/multi-layer graphene is prone to cracks or discontinuities. HNO_3_ doping addresses this issue, but impact on biocompatibility must be further studied.	Such thin layers of Au have discontinuities. Transparent layers of PIE/PSS are used to address this issue.	Stability of modified PEDOT:PSS electrodes is uncertain based on previously shown delamination failures of electrodeposited PEDOT:PSS electrodes [30].

^a^ Reported in this manuscript.

Considering all these trade-offs, ITO was selected as the transparent conductive material to implement the transparent Smart Dura. This material could be readily used in our fabrication process, which was already optimized for the previous generation of Smart Dura. The drawback of ITO’s brittleness is overcome through design implementations that reduce the length of ITO traces and mainly use ITO in the center of the device, where mechanical bending during handling, implantation and post-implantation is minimal [19].

In this paper we present the design, fabrication, and benchtop characterization results of our Smart Dura with transparent ITO electrodes in addition to *in vivo* demonstration of multimodal interfacing with NHP brain.

## 2. Methods

### 2.1. Design and fabrication of transparent Smart Dura

#### 2.1.1. Design

Our legacy Smart Dura was designed with micro-size metal features and a honeycomb electrode lattice to provide uniform, large-scale coverage, high-resolution electrophysiology with simultaneous optical access [18, 19]. Building on the success of our legacy Smart Dura platform, the design of our transparent Smart Dura preserves some design features while also adopting new ones for the successful incorporation of transparent ITO electrodes. This time, as shown in figure 1(a), we designed seven NHP transparent Smart Dura arrays with 64-, 128- and 192-channels. In these designs, the electrodes are also arranged uniformly distributed with a honeycomb lattice to maximize electrode coverage for a 20 mm diameter area (314 mm^2^). The electrode pitches selected for these designs are 1.5 mm, 1 mm and 0.75 mm respectively. The electrode dimensions were also chosen for high resolution electrophysiological recording with 20, 30 and 40 *µ*m electrode diameters (shown in figure 1(e)), which are similar in size to the cell body of cortical neurons. Additionally, we designed one 63-ch device with considerably larger electrode dimensions (400 *µ*m diameter), shown in figure 1(d), to discern the electrochemical and electrophysiological differences between large electrodes and those typical of microelectrode arrays. Besides the aforementioned design parameters, the most relevant design feature for the transparent Smart Dura is found in the implementation of ITO electrodes. As mentioned in the introduction, the ideal approach of making electrodes and traces entirely out of ITO is not a plausible option due to cracks in the ITO film upon bending forces. Instead, our implementation of ITO consists of using ITO for all geometrical features greater than 10 *µ*m wide (electrode sites and tapered geometries used for routing) with the rest of the routing traces using Pt/Au metal stack and an overlap region of ITO + Pt/Au metal stack to guarantee electrical continuity, as shown in figures 1(b), (c), and (f). This design is similar to the *µ*ECoG design reported in Ledochowitsch *et al* [31] for rodents. Note that the trace width of the ITO geometry in the overlap region is slightly wider (14 *µ*m) than the 10 *µ*m Pt/Au trace to ensure proper adhesion to the Pt/Au metal stack and avoid delamination during subsequent fabrication steps.

**Figure 1.**
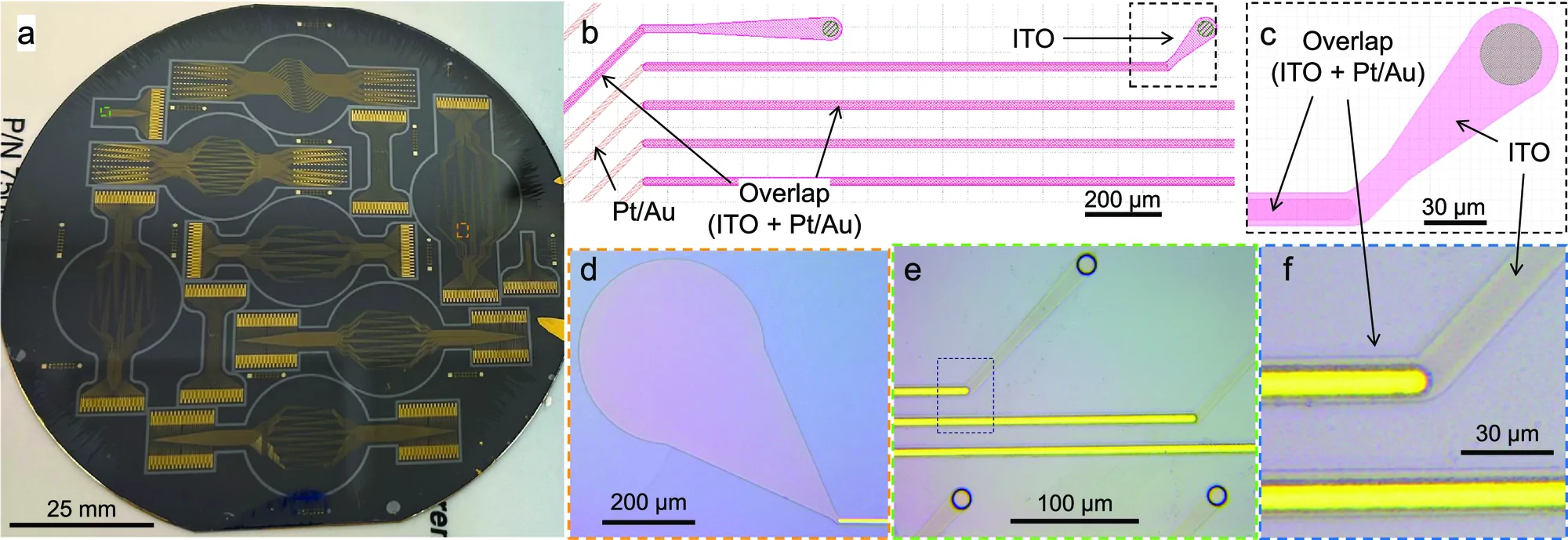
Transparent Smart Dura design and device features. (a) 4-in wafer containing various designs of our transparent Smart Dura before the release step. (b) Design of the indium tin oxide (ITO) electrodes and routing traces of our transparent Smart Dura with the implementation of an ITO + Pt/Au metal stack overlap regions, with a zoomed-in inset of the electrode region in (c). (d) Micrograph of our large 400 μm diameter ITO electrode. (e) Micrograph of 20 μm diameter ITO electrodes and traces. Inset in (f) shows the fabrication results of the ITO + Pt/Au metal stack overlap region providing continuous contact for signal routing.

With this ITO-Pt/Au hybrid approach we are able to: (i) address the mechanical limitations of ITO material properties (brittleness) by reducing the length of the ITO structures and confining it to the center of the device where bending forces are minimum for the application, (ii) minimize the resistivity of the overall routing trace by leveraging the higher conductivity of Au to carry the signal for most of the length of the trace and (iii) provide a field of view into the surface of NHP brain with 100% effective optical access, which we previously defined in [19], enabled by the 10 *µ*m wide Pt/Au traces and ITO electrodes.

#### 2.1.2. Fabrication

The fabrication of the Smart Dura with transparent electrodes was adapted from our legacy fabrication process [19] to incorporate ITO films into the material stack via sputtering deposition. Given the high yield throughput of our legacy fabrication process, we maintained the conservative 10 *µ*m trace width resolution as our minimum feature for our NHP devices, despite our process having the capability of 1 *µ*m feature resolution. The microfabrication process is outlined in figure 2(a).

**Figure 2.**
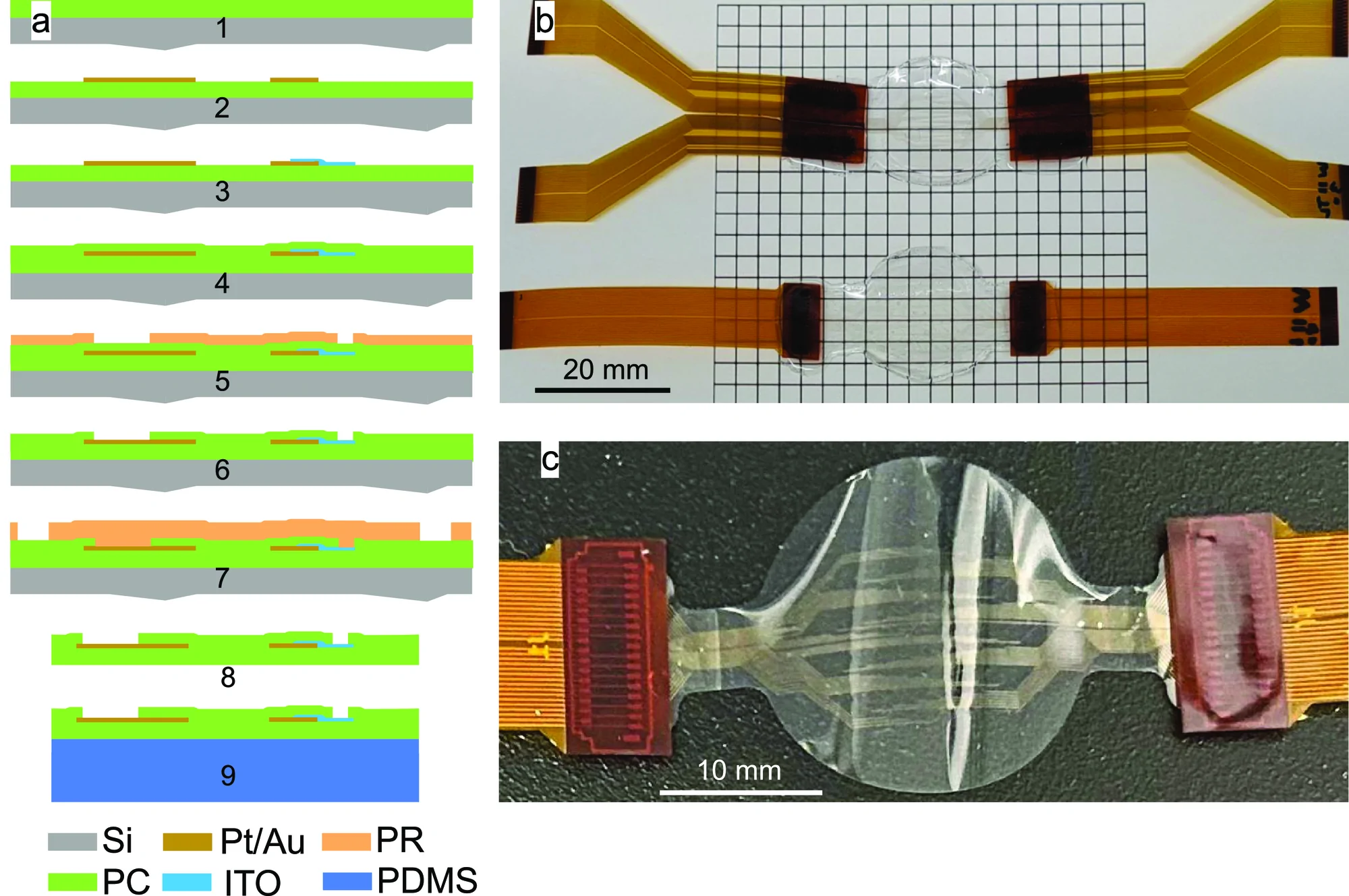
Transparent Smart Dura fabrication process flow, packaging and optical access. (a) Microfabrication process illustrating the layer-by-layer transition in each fabrication step: (1) conformal deposition of Parylene C (PC) substrate, (2) Pt/Au metal stack deposition and patterning via lithography, metal deposition and lift-off for traces and contact pads, (3) ITO deposition and patterning via image reversal lift-off for transparent electrodes, (4) Parylene C conformal deposition for the insulation layer. Parylene C is etched in two steps using a thick photoresist (PR) soft mask (5) to expose electrodes and contacts (6) and define the device outline (7). The last steps of the fabrication process are the release of the device from the Si carrier wafer (8) and the adhesion of a polydimethylsiloxane (PDMS) backing layer to the substrate of the Smart Dura (9). (b) Packaging of 64-ch and 128-ch transparent Smart Dura with flexible printed circuit board (PCB) containing zero insertion force (ZIF) connector(s) to extend routing and provide more mechanically robust interconnects. (c) Highly transparent optical access of our transparent Smart Dura enabled by ITO electrodes and practically transparent 10 *µ*m metal traces.

The fabrication process starts with a bare silicon wafer as the temporary substrate. A 5 *µ*m thick layer of Parylene C was deposited using chemical vapor deposition (Labcoter 2, Specialty Coating Systems, Indianapolis, IN) (1). The benefit of depositing Parylene C directly on Si is that there is no need for a sacrificial layer. The adhesion of Parylene C to Si is strong enough to withstand the changes in temperature and pressure as well as exposure to chemical solvents during the various steps of the fabrication process without delamination but is not strong enough that a diluted Micro 90® solution [32] can be used as a release agent. After Parylene C deposition, the 10 *µ*m wide electrical traces were defined using photolithography. A 1.5 *μ*m thick image reversal (AZ5214E) photoresist was patterned using contact lithography (MA6 contact aligner, Karl Süss, Garching, Germany). Following the development in AZ400K (MicroChemicals), the wafer surface was cleaned in IPA and deionized (DI) water, and the exposed Parylene C was activated using O_2_ plasma in Trion RIE (Phantom RIE, Trion, Clearwater, FL) at 100 W and 100 mTorr for 30 s. Pt (10 nm)/Au (100 nm) was deposited on the patterned photoresist by electron-beam evaporation (Kurt J. Lesker PVD 75 Electron Beam Evaporator, Jefferson Hills, PA). Excess metal was lifted off in an acetone bath for two hours plus successive rinses were done in IPA and DI water for 5 min (2). For the remainder of the routing (tapered geometries) and electrode sites, the same photolithographic and lift-off process was repeated but with ITO reactive sputtering deposition from a In_2_O_3_/SnO_2_ 90/10 wt % target via reactive sputtering in a O_2_ + Ar plasma (LGA Thin Films, Santa Clara, CA). Once again, excess metal was lifted off in an acetone bath for two hours followed by successive rinses in IPA and DI water for 5 min (3). After the two lift-off processes, another 5 *µ*m layer of Parylene C was conformally deposited as the top electrical insulation layer (4). In order to remove the Parylene C at the electrode sites, contacts, and device outline, a thick layer (12 *µ*m) of photoresist (AZ4620) was deposited and patterned and acted as a protective soft-mask layer (5). Then the Parylene C was etched using a cycled O_2_ plasma reactive ion etching (RIE) process (3 min etch and 2 min rest) to expose the electrode sites and the contact pads (6) in the Trion RIE (Phantom RIE, Trion, Clearwater, FL). During the last cycles of etching, the etched trenches were measured using a surface profilometer (P-15 Stylus Profiler, KLA Tencor, USA), to confirm electrodes and contact pads were fully exposed. Because the thick photoresist layer has the same etch rate as Parylene C to O_2_-based RIE, profilometer measurements and optical microscopy inspection were used to determine that all electrodes and contact pads were fully exposed. While in some instances using a soft-mask layer can pose a risk of etching unwanted regions, the etch rate in our process (0.5 *µ*m min^−1^) and the thickness of the photoresist layer (more than double the thickness of our Parylene C insulation layer) provides enough margin in the etching tolerance that the risk of unwanted etching is practically eliminated. This can be seen in figure 1(a) by noting the undisturbed Parylene C insulation layer on the Smart Dura devices after all O_2_ RIE was performed. Next, to remove the Parylene C at the device outline the same procedure was followed. In this case the layer of photoresist was thicker (24 *µ*m) by spin coating two thick layers of AZ4620 (12 *µ*m each) to protect the exposed electrodes and contact sites and only the outline region was exposed (7). Lastly, the exposed Parylene C was etched via the same O_2_ plasma RIE process and the device was released from the Si wafer in a DI water: Micro-90® solution (5:1 by weight) after mechanically pulling at the edge of the device using metal tweezers (8). After release and packaging with flexible PCBs, a 200 *µ*m thick polydimethylsiloxane (PDMS) backing layer cast separately, was attached to the back of each released device using a very thin and uncured PDMS diluted in hexane (10:1 by weight hexane:PDMS solution) that acted as glue (9).

Exemplary microfabricated NHP transparent Smart Dura devices (64- and 128-channel designs) are shown in figures 2(b) and (c). As shown, device packaging for the 64- and 128-channel designs was performed with zero-insertion-force (ZIF) connectors using compatible pad arrays located in the *arms* of the device. For the 192-channel design, we designed a bond pad array compatible with anisotropic conductive film bonding or bump bonding for a more compact packaging footprint [33, 34]. As with the legacy Smart Dura devices, we connected the backend connector pads of the transparent Smart Dura devices to polyimide-based flexible cables integrated with ZIF connectors and attached the 200 *µ*m thick backing layer of PDMS to provide easier and more robust connectivity to the back-end electronics prior to *in vivo* implementation. The successful fabrication of the transparent Smart Dura with ITO electrodes, as shown in figure 2(c), improves the optical properties of our legacy Smart Dura by providing a field of view to the surface of NHP brain with 100% effective optical access enabled by the 10 *µ*m wide Pt/Au traces and ITO electrodes.

### 2.2. Electrical impedance spectroscopy (EIS)

For the EIS measurements, we used a potentiostat system (Autolab, PGSTAT204, Metrohm Co) in a three-electrode electrochemical cell configuration. The device was immersed in a phosphate-buffered-saline (PBS) as described in our previous publication [19]. In short, the impedance of the transparent Smart Dura ITO electrodes was obtained by the voltage and current measurements from the Ag/Ag–Cl reference electrode and the tungsten counter electrode, respectively.

### 2.3. Noise analysis

Additionally, the electrochemical impedance values obtained from the EIS measurements provide a baseline signal-to-noise-ratio (SNR) level of our electrodes by considering its intrinsic thermal noise. The theoretical SNR of our electrodes can be computed from equation (1) below,

\begin{equation*}SN{R_{{\mathrm{dB}}}} = 20{\mathrm{log}}\left( {\frac{{{V_{{\mathrm{signal}}}}}}{{{V_{{\mathrm{noise,rms}}}}}}} \right).\end{equation*}

The noise term in the denominator is obtained from the thermal noise model of a resistive device provided by equation (2):

\begin{equation*}{V_{{\text{noise, rms}}}} = \sqrt {4{k_{\mathrm{B}}}{ }T{ }R{ }\Delta f} ,\end{equation*}

where *k*_B_ is Boltzmann constant (1.38 × 10–23 J K^−1^), T is temperature (300 K), *R* is the real part of our electrode impedance (*R* = |*Z*| cos*θ*, where |*Z*| and *θ* are the impedance amplitude and phase) and Δ*f* is the frequency bandwidth. For the electrodes of the Smart Dura (and more broadly for electrodes used in electrochemical applications) the impedance is frequency dependent. Thus, the resistance needs to be integrated across the frequency band of interest and equation (2) is modified accordingly as shown in equation (3) below, as

\begin{equation*}{V_{{\text{noise, rms}}}} = \sqrt {4{k_{\mathrm{B}}}{ }T\mathop \int \limits_{{f_{{\mathrm{low}}}}}^{{f_{{\mathrm{high}}}}} R{\text{ d}}f.} \end{equation*}

We validated the noise floor of the ITO electrodes by performing baseline electrophysiological recording using the Intan RHD2000 system with 40 *µ*m diameter Smart Dura ITO electrodes on 1x PBS solution. Using the Intan RHX data acquisition software, we performed baseline recordings for noise measurements for 10 min. The average root-mean-square (RMS) noise level was calculated using equation (4) below:

\begin{equation*}{V_{{\text{ rms, total}}}} = \sqrt {\frac{1}{N}\mathop \sum \limits_{n = 1}^N x_n^2} \end{equation*}

where ${x_n}$ are the filtered samples.

### 2.4. Cyclic voltammetry (CV)

Using the same three-electrode electrochemical cell as in EIS, we performed CV to expand the electrochemical characterization of the ITO electrodes of the transparent Smart Dura. In this method, the potential of the working electrode is cyclically swept at a constant rate between two voltage limits, relative to the Ag/AgCl reference electrode that does not carry current. Thus, current flows between the working and counter electrode. The applied potential drives electrochemical reactions at the working electrode, and the resulting current reflects the rate of these reactions. We performed CV by applying a square-stepped voltage waveform swept cyclically between −0.55 V to 1.8 V for 5 cycles at a scan rate of 50 mV s^−1^. The voltage range (−0.55:1.8 V) lies within the electrochemical window of water (ensuring no oxidation or reduction of the solvent) for ITO electrodes as we previously determined (figure 5(a)).

### 2.5. High spatial resolution optical spectroscopy

We performed UV-Vis-IR spectroscopy using the optical setup shown in figure 6(a) to characterize the optical transmission of the materials used in the transparent Smart Dura with micrometer-scale spatial resolution. In brief, broadband illumination was focused onto the sample to form a localized spot (<30 *µ*m in diameter), enabling spatially resolved transmission measurements. Transmitted light was collected beneath the sample and analyzed spectrally to quantify attenuation across the visible and near-infrared range. To account for background contributions and source intensity variations, transmission values were calculated using dark and reference measurements. Measurements were performed for the different transparent films of the transparent Smart Dura: PDMS, Parylene C and ITO. For the isolated measurement of ITO films, we used glass slides samples where we sputtered 100 nm of ITO directly. A detailed description of the optical setup, calibration procedure, and data processing workflow is provided in our previous publication [18].

In addition to optical transmission, we also characterize the optical performance of the Smart Dura through its optical access. As we described in our previous publication, optical access is the percentage of the total Smart Dura field of view (FOV_total_) not blocked by opaque metallic features [19]. In mathematical terms, optical access (abbreviated as OA in the mathematical expression) is defined by equation (5) below:

\begin{equation*}{\mathrm{OA}} = \frac{{{A_{{\mathrm{op}}}}}}{{{\mathrm{FO}}{{\mathrm{V}}_{{\mathrm{total}}}}}} \times 100,\end{equation*}

where *A*_op_ is the geometrical area occupied by opaque metal traces and electrodes within the field of view (FOV_total_) of the Smart Dura.

As discussed in our previous publication, considering the practical constraints where the smallest optical target has an average size of 20 *µ*m in diameter (i.e. a pyramidal cell), the 10 *µ*m wide metal traces of the Smart Dura have a minimal impact on optical acces. Thus, we define the term effective optical access as the percentage of the total Smart Dura field of view (FOV_total_) not blocked by opaque metallic features wider than 10 *µ*m [19]. In mathematical terms, effective optical access (OA_eff_) is defined by equation (6) below:

\begin{equation*}{\mathrm{O}}{{\mathrm{A}}_{{\mathrm{eff}}}} = \frac{{{A_{{\mathrm{op}} &gt; 10{ }\mu m}}}}{{{\mathrm{FO}}{{\mathrm{V}}_{{\mathrm{total}}}}}} \times 100,\end{equation*}

where *A*_op > 10 *µ*m_ is the geometrical area occupied by opaque metal features (traces and electrodes) wider than 10 *µ*m.

### 2.6. Animals and Smart Dura placement

Animal care and experiments were approved by the University of Washington’s Office of Animal Welfare, the Institutional Animal Care and Use Committee, and the Washington National Biomedical Research Center (WaNBRC). The 63-ch and 128-ch transparent Smart Dura were tested in a female macaque: Macaca nemestrina (6 kg, 5 years). The animal underwent a 25 mm diameter craniotomy and durotomy targeting the V1 in the left hemisphere. A 3D-printed holder for securing the Smart Dura and recording components was fixed to the skull using dental acrylic, and the Smart Dura was then positioned on the cortical surface, as described in previous studies [18, 19]. Throughout the experiment, the animal was anesthetized with a continuous infusion of sufentanil citrate (4–6 *µ*g kg^−1^ h^−1^) and vecuronium bromide (initial bolus of 0.1 mg kg^−1^; 0.1 mg kg^−1^ h^−1^). The details of our surgical procedure are described here [10].

### 2.7. Optical coherence tomography angiography

A custom-built swept-source optical coherence tomography system was employed to perform OCTA imaging [35]. The system utilized a swept-source laser operating at a 200 kHz A-scan rate, with a central wavelength of 1310 nm and an optical bandwidth of 100 nm, yielding an axial resolution of approximately 8 *µ*m in tissue. An objective lens (LSM04; Thorlabs) with a focal length of 54 mm provided a lateral resolution of approximately 35 *µ*m. The total optical power incident on the Smart Dura was 5 mW. Each OCT volumetric acquisition required 90 s and covered a 20 × 20 mm^2^ cortical area. Volumes were raster-scanned into 1200 cross-sectional planes, with each plane consisting of 1200 A-scans. To enable blood flow contrast, 12 repeated B-scans were acquired at each individual plane. A system-integrated RGB camera was used for real-time navigation of the imaging probe. OCTA image processing was performed in Python. First, an Eigen-decomposition–based algorithm was applied to extract blood flow contrast, using four repeated B-scans per batch, resulting in three batches at each spatial location. Subsequently, a motion-rejection algorithm was employed to discard frames exhibiting severe cardiac and respiratory motion artifacts. The remaining batches from the same location were then averaged to improve signal robustness. Finally, top-down consecutive maximum-intensity projection was applied to collapse the three-dimensional blood flow volumes into two-dimensional vascular maps, with pseudo-color encoding used to represent vessel depth.

### 2.8. Surface imaging

Brightfield imaging of the surface of the NHP cortex was performed with a sCMOS camera (Zyla-4.2 USB3; Andor) equipped with a tandem lens configuration (Nikkor 50 mm and Nikkor 85 mm; Nikon) to achieve a 22.6 mm field of view (figures 7(c) and (d)). The zoomed-in images to visualize the ITO electrodes on top of NHP cortex (figures 7(e) and (f)) were acquired using a digital camera (EOS Digital Rebel T31, Canon) mounted to the wide-field path of the multi-photon microscope.

### 2.9. Multiphoton imaging

The details of two-photon imaging procedures for the legacy Smart Dura are described here [18, 19]. Briefly, two-photon fluorescence imaging of cortical vasculature was performed under anesthesia, using intravenous fluorescent agent FITC-dextran and standard multiphoton microscopy to visualize blood vessels through the legacy Smart Dura. Three-photon imaging was performed through transparent ITO electrodes using a multi-photon microscope (MOM; Sutter Instruments, CA) equipped with an excitation laser set to 1300 nm wavelength (Monaco/Opera-F; Coherent) and a 25x water immersion objective lens (XLPLN25XWMP2; NA = 1.05; Olympus, Japan). Following the Smart Dura placement, a custom 3D-printed imaging well with a glass bottom was fixed on the Smart Dura for immersion of the objective lens, and FITC-dextran (50 mg kg^−1^; 50 mg ml^−1^ in sterile water; 52 471; Sigma–Aldrich, MA) was then intravenously injected [18]. 3D image stacking, pseudo-coloring, and intensity profile measurements were performed using MATLAB (MathWorks, MA).

### 2.10. Visual stimulation

The details for the visual stimulation experimental paradigm are identical and described in [18]. Briefly, visual responses were evoked using drifting chromatic and achromatic grating stimuli presented monocularly in multiple directions, and neural activity was quantified by time–frequency analysis of electrophysiological recordings to extract high-gamma (HG) power.

### 2.11. Electrophysiological recording with visual stimulation

To record electrophysiological signals, the Smart Dura was first connected to front ends (Nano-HV and Nano2 + Stim; band-pass filter: 1 Hz–7.5 kHz) and then secured onto the holder, and the grounding pins were connected to a skull screw that was positioned anterior to the craniotomy. Signal acquisition was controlled by a Grapevine Nomad processor and Trellis software (Ripple Neuro, UT). LFPs were sampled at 1 kHz and digitally filtered with a low-pass filter at 250 Hz, with notch filters at multiples of 60 Hz. To examine the evoked response to visual stimulation, a multitaper spectrogram (300-ms time window with 3-ms steps) was generated and z-scored to the frequency of band power 4 s preceding the stimulus. The average HG power was obtained by extracting the bins of 60–150 Hz from the z-scored spectrograms and averaging across trials. All signal processing and data analyses were carried out using MATLAB (MathWorks, MA).

### 2.12. Wide-field ISOI in response to visual stimulation

The imaging system and image acquisition process are the same as described in [18]. Cortical activity was recorded using wide-field optical imaging through the Smart Dura under LED illumination, and image sequences were analyzed to quantify stimulus-evoked changes relative to a pre-stimulus baseline. Functional color domains and ocular dominance maps were generated by contrasting responses across stimulus conditions, while vasculature images acquired with the same setup were used to align electrode positions. For comparison with electrophysiological measurements, intrinsic signal time courses were extracted from regions adjacent to selected electrodes and averaged.

For the analysis of the ISOI signal, the response amplitude was calculated as a fraction of the image intensity from the baseline. First, baseline intensity *F*_0_ was calculated as an average over 1 s before the stimulus onset. Then, *F*(*t*) was calculated as an average with a shifting time window of a 1 s width. Then, the amplitude was calculated as Δ*F*(*t*)/*F*_0_ = (*F*(*t*)-*F*_0_)/*F*_0_. The ocular dominance pattern was visualized by comparing the left eye and the right eye conditions. Alignment of transparent ITO electrodes was performed in KLayout (Matthias Koefferlein, Germany) to match the visible landmarks of the Smart Dura metal features in the image (figure 10(a)) to the design file. For the comparison with HG power measured by the Smart Dura, time courses of the intrinsic responses were obtained from 7 × 7 pixel grid beneath the electrode and averaged (figures 10(e) and (f)). Similarly, the time courses of the intrinsic responses for figure S3 and S5 were obtained from 1 pixel (figure S3(b)–(d)), and the average ISOI trace of various size pixel grids (3 × 3, 7 × 7, 15 × 15, 31 × 31, 39 × 39, in figure S3 (e)–(i) and 13 × 13, 13 × 13 with the central 9 × 9 pixels excluded in figure S5).

## 3. Results

### 3.1. Electrochemical and Optical Characterization

After microfabrication and packaging, the electrochemical and optical functionalities of our Smart Dura with transparent ITO electrodes were characterized to evaluate the yield in fabrication and the electro-optical performance prior to *in vivo* validation. EIS and CV were used to assess the electrochemical characteristics of ITO electrodes for electrophysiological recording and electrical stimulation. It is worth pointing out that finding available publications in literature that characterize the electrochemical properties of ITO electrodes of different sizes used in neural interfaces is limited, and unfortunately the data found in relevant publications is often incomplete. Here we provide a rigorously and thoroughly measured EIS, baseline noise and CV data set for ITO electrodes with diameters of 20, 30, 40, and 400 *µ*m. Additionally, high resolution optical spectrometry was conducted to characterize the optical transparency of the ITO films integrated into our transparent Smart Dura.

#### 3.1.1. EIS

Following the standard three-electrode electrochemical cell on 1x PBS solution for EIS [36], we measured the electrochemical impedance on each of our devices for bench-top assessment on the electrophysiological recording performance of ITO electrodes. As shown in figures 3(a) and (b), the electrochemical response vs frequency of the various size ITO electrodes closely follows a similar trend (with some scaling factor based on size) except for the 400 *µ*m diameter electrode at higher frequencies (>10 kHz). For frequencies <10 kHz, despite the difference in electrode size, the electrode-electrolyte interface is highly capacitive, with impedance phase values < −60°, indicating that the electrochemical transduction mechanism is governed by capacitive coupling rather than faradaic coupling [37].

**Figure 3.**
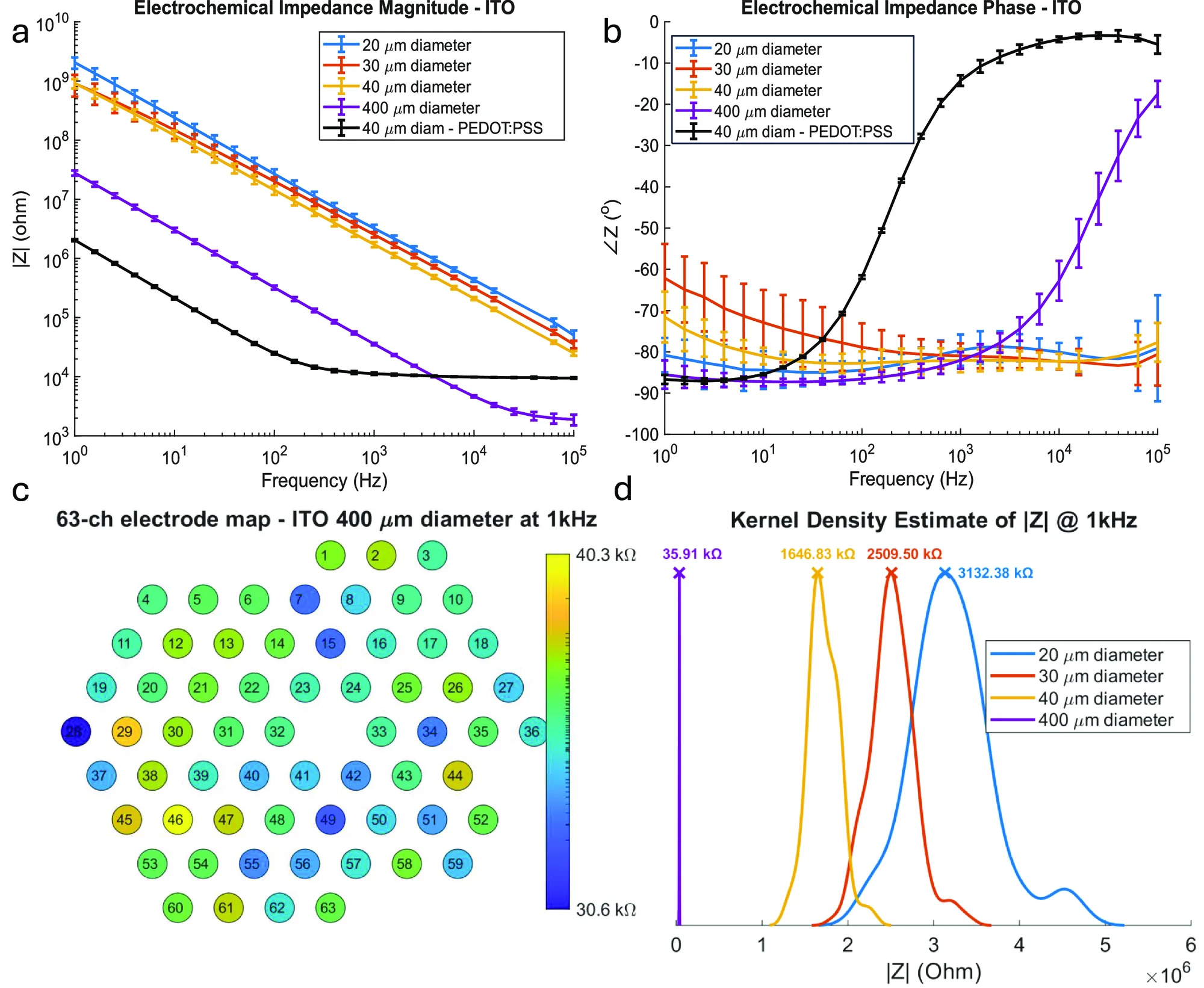
Electrochemical Impedance Spectroscopy characterization of ITO electrodes. Electrochemical impedance magnitude (a) and phase (b) vs frequency of different size ITO electrodes. Note the closely similar trend in impedance amplitude and phase across the different electrode sizes for most frequencies (except 400 *µ*m diameter electrodes at frequencies higher than 10 kHz) indicating that the electrode-electrolyte interface is highly capacitive, with impedance phase values <−60°, and that the electrochemical transduction mechanism is governed by capacitive coupling rather than faradaic coupling [37]. (c) Electrode map of the 63-ch transparent Smart Dura with 400 *µ*m diameter ITO electrodes with impedance values at 1 kHz representing the high yield of our fabrication and packaging processes and the spatial distribution of impedance values with location. (d) Kernel Density Estimate of the impedance magnitude at 1 kHz of our transparent Smart Dura devices with different electrodes sizes. Note the narrow deviation in the impedance distribution demonstrating the consistency in electrode impedance and the low noise deviation across our ITO electrodes. The sample size (*n*) of the 20, 30, 40, and 400 *µ*m diameter ITO electrodes is *n*= 64, *n* = 121, *n* = 64, and *n* = 63, respectively. The data for each ITO diameter is from electrodes across the same device.

The electrochemical impedance magnitude (|Z|) of the ITO microelectrodes at the frequencies of interest of neural interfaces (<100 kHz) is high compared to other electrode material counterparts (i.e. Pt, Au, PEDOT:PSS) which is consistent with its highly capacitive response. At 1 kHz, the impedance magnitude for 20, 30, and 40 *µ*m diameter electrodes is 3.22 ± 0.48 MΩ (*n* = 64), 2.52 ± 0.26 MΩ (*n* = 121), and 1.72 ± 0.18 MΩ (*n* = 64), respectively. In contrast, the impedance magnitude for 400 *µ*m diameter electrodes is 35.91 ± 1.68 kΩ (*n* = 63). The small deviation of impedances at all measured frequencies as well as the normal distribution of impedance magnitudes at 1 kHz (with very few outliers) shown in the kernel density estimate across the ITO electrode arrays of different electrode sizes (figures 3(c) and (d)) confirm the high yield within a fabrication run of the microfabrication and packaging processes. This also shows that the variation of impedance is less than 15% across all the electrodes for 20 *µ*m, around 10% for 30 and 40 *µ*m and less than 5% for 400 *µ*m diameter electrodes. Such low variation in impedance provides homogeneous noise floor levels across the entire ITO electrode array for uniform electrophysiological recording. In other words, the observed impedance variation is not large enough to necessitate calibration across the electrode array. Additionally, the total yield of all the fabricated and packaged transparent Smart Dura devices (20 total devices) across four fabrication runs was 90.19%.

#### 3.1.2. Noise characterization

It is often deemed necessary to obtain an impedance magnitude lower than 1 MΩ, citing signal-to-noise ratio requirements for proper electrophysiological recording [36], or low input impedance of common neural amplifiers (10.6 MΩ at 1 kHz for the Ripple Neuro system [38] and 13 MΩ at 1 kHz for the Intan system [39]). However, despite the high impedance of the ITO microelectrodes (>1.5 MΩ), computing the thermal noise (see methods for more details) of the ITO electrodes for the LFP frequency band (10–250 Hz) using equation (3) reveals low noise levels of 3.60 ± 0.43 *µ*V_rms_ (*n* = 64), 4.50 ± 0.76 *µ*V_rms_ (*n* = 121), 3.09 ± 0.28 *µ*V_rms_ (*n* = 64) (figure 4(a)), and 0.31 ± 0.03 *µ*V_rms_ (*n* = 63) for 20, 30, 40, and 400 *µ*m diameter electrodes respectively. These noise levels are within the same order of magnitude as those from gold microelectrodes of similar size. Unexpectedly, we observed that the thermal noise level of the 20 *µ*m diameter ITO electrode is lower than the noise of the 30 *µ*m diameter ITO electrode. A small change in the impedance phase of ∼10 degrees in the LFP band (figure 3(b)), to a more resistive behavior is responsible for this effect on the thermal noise. The thermal noise for the spike frequency band (0.25–10 kHz), is 6.05 ± 0.74 *µ*V_rms_ (*n* = 64), 4.96 ± 0.62 *µ*V_rms_ (*n* = 121), 3.85 ± 0.49 *µ*V_rms_ (*n* = 64) (figure 4(a)), and 0.70 ± 0.06 *µ*V_rms_ (*n* = 63) for 20, 30, 40, and 400 *µ*m diameter electrodes respectively. The comparable or greater thermal noise level observed in the wider spike-band bandwidth, relative to that of the LFP band, indicates that the increased resistive contribution to the thermal noise in the LFP band is effectively equivalent to the contribution arising from the total integrated bandwidth of the spike band. Note that despite a higher impedance of almost 3 orders of magnitude difference between 20 *µ*m and 400 *µ*m diameter ITO electrodes, the thermal noise in both frequency bands is only 1 order of magnitude higher and considerably lower than typical electrophysiological signal amplitudes values.

**Figure 4.**
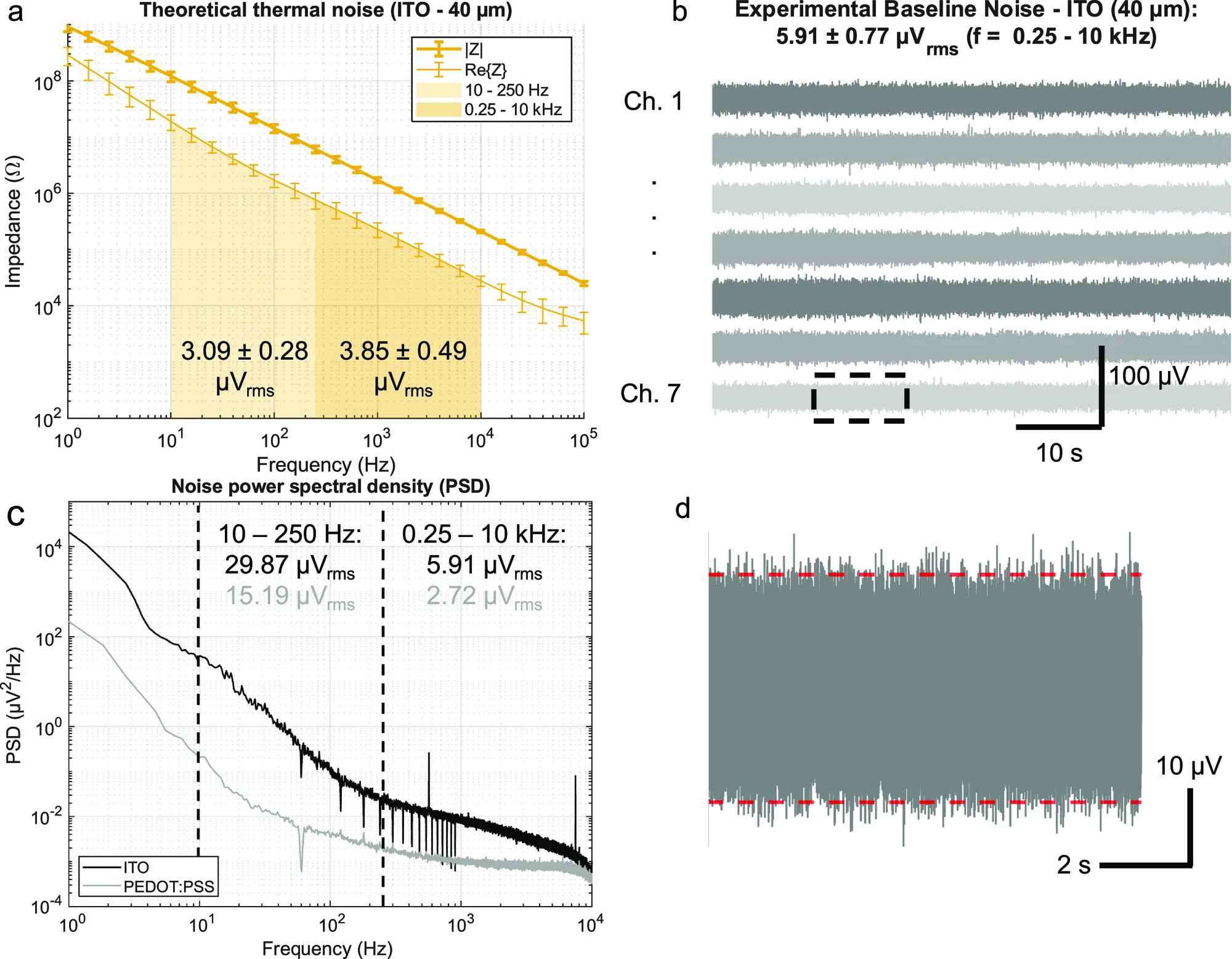
Baseline noise level of 40 *µ*m diameter ITO electrodes on 1x phosphate-buffered saline (PBS) solution. (a) Impedance magnitude (|Z|), real component (Re{Z}) of the 40 *µ*m diameter ITO electrodes, and theoretical thermal noise values for the local field potential (LFP) frequency band (10–250 Hz) and spike frequency band (0.25–10 kHz) (*n* = 64). (b) Voltage levels of 7 different ITO electrodes across a 50 s recording. (c) Power spectral density of a selected ITO channel showing the additional frequency dependent noise components present in the baseline time-domain recording and a direct comparison with an equal sized PEDOT:PSS electrode. (d) Zoomed in figure of the voltage signal for an individual electrode. The red dash lines show the 99.9th percentile of the voltage.

We validated the noise floor by performing baseline electrophysiological recording using the Intan RHD2000 system with 40 *µ*m diameter Smart Dura ITO electrodes on 1x PBS solution. Representative baseline recordings of seven channels are shown in figure 4(b) after filtering the raw signal to the frequency range of 0.25–10 kHz, with figure 4(d) showing a close-up view of the baseline signal level with the red dash lines indicating the 99.9th percentile of the voltage signals, illustrating the noise floor of the ITO electrodes for the spike band. From these measurements, the average RMS noise level was calculated using equation (4). The total RMS noise mean and standard mean error (*n* = 26) for the 10–250 Hz frequency range was 29.87 ± 4.20 *µ*V_rms_ and for the 0.25–10 kHz frequency range was 5.91 ± 0.77 *µ*V_rms_. These results are higher than the thermal noise values previously computed. The discrepancy between the thermal noise calculated from the EIS measurements and the total noise in the electrode can be explained by the presence of other sources of noise present during the baseline recording, such as flicker noise, also known as 1/f noise, and Brownian noise, also known as 1/f^2^ noise. Figure 4(c) shows the power spectral density of the raw trace from a selected channel showing precisely the additional frequency dependent noise. Both flicker and Brownian noise are associated with electrode–electrolyte interface processes and material properties. These components decrease with frequency and become negligible in the high-frequency band (⩾10 kHz), where the noise spectrum approaches the flat white noise floor, primarily determined by thermal noise and amplifier input noise.

Computing the estimated SNR values (equation (1)) for LFP and single-unit spikes, the SNR would be at a minimum of 20.03 dB, considering a 300 *µ*V signal from an LFP signal and the measured noise of 29.87 *µ*V_rms_ at the frequency range 10–250 Hz, and at a minimum of 28.09 dB, considering a 150 *µ*V signal from a single-unit and the measured 5.91 *µ*V_rms_ at the frequency range 0.25–10 kHz.

From these measurements, we demonstrated that the noise floor of the ITO electrodes of the transparent Smart Dura devices is low and within the same order of magnitude as the noise floor of the electrophysiological recording equipment (2.1 *µ*V_rms_ at 1 kHz for the Ripple Neuro system [38] and 2.4  *µ*V_rms_ at 1 kHz for the Intan system [39]) and consistent across all working electrodes, given the small standard mean error.

#### 3.1.3. CV

In addition to impedance measurements, we performed CV using the same three-electrode electrochemical cell we used for EIS. The primary goal was to quantify the charge storage capacity (CSC) of the Smart Dura ITO electrodes; a metric used to evaluate the safety and efficacy of electrodes for electrical stimulation. CSC reflects the electrode’s ability to reversibly inject charge before exceeding the water electrolysis window, which directly impacts stimulation performance and long-term stability. CV is an appropriate technique for this purpose because it not only provides the integrated charge (CSC and cathodal CSC_c_) but also offers insight into electrochemical activity, reaction reversibility, and electrode integrity.

The low levels of current obtained during CV provide additional evidence of the capacitive coupling mechanisms of ITO microelectrodes and absence of faradaic charge transfer mechanisms. As shown in figure 5(a), the voltage range before inducing the electrolysis of water, known as the water window, for ITO electrodes is −0.55–1.8 Volts. Beyond these voltage levels, an exponentially increasing current–voltage response follows from chemical reactions involving water molecules in the PBS solution. These reactions cause the release of ions into the electrolyte with adverse effects such as subsequent chemical reactions or changes in pH levels in biological tissue.

**Figure 5.**
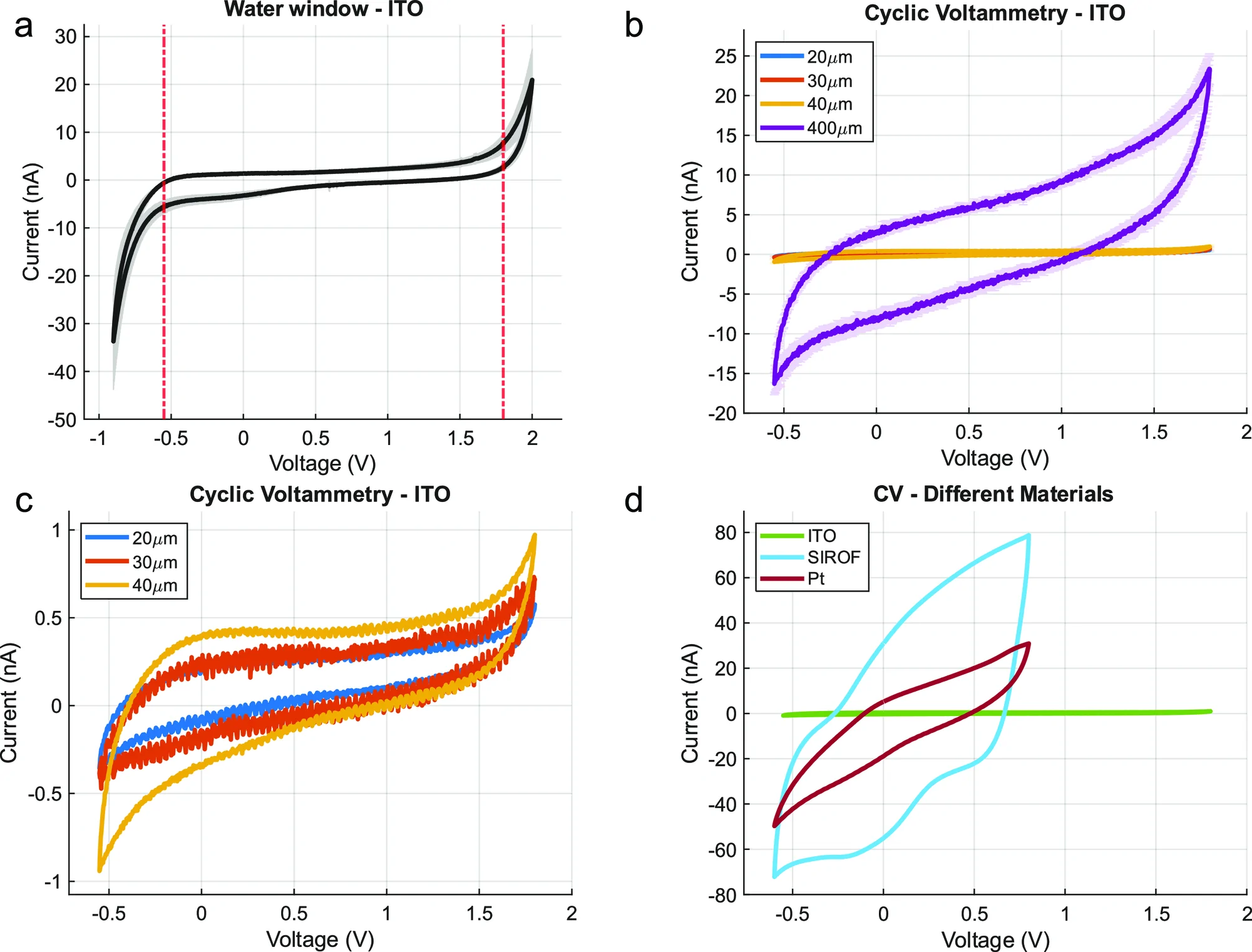
Cyclic voltammetry of ITO electrode. (a) Cyclic voltammetry (CV) exceeding the voltage levels to induce electrochemical reactions of ITO with the water based 1x PBS solution to clearly observe the exponential increase in current levels due to the hydrolysis of water (*n* = 52). Note the high cathodic voltage level before reaching the knee of the exponential current increase, yielding a water window between −0.55 and 1.8 V, as shown by the red vertical lines. (b) CV scans within the water window of ITO for 20, 30, 40, and 400 *µ*m diameter electrodes. The sample size (*n*) of the 20, 30, 40, and 400 *µ*m diameter ITO electrodes for the CV data is *n* = 63, *n* = 7, *n* = 57, and *n* = 13, respectively. The data for each ITO diameter is from electrodes across the same device. Note the small amounts of current drawn within the water window, confirming capacitive coupling as the dominant charge-exchange transduction mechanisms of ITO. (c) Zoomed in CV plots of 20, 30, and 40 *µ*m diameter electrodes showing the mean response. (d) Comparison between CV scans of ITO, sputtered iridium oxide film (SIROF), and Pt, each within their own water window, showing the low charge storage capacity capability of ITO compared with other electrode materials more commonly used for neural electrical stimulation. This confirms the role of ITO electrodes as primarily used for electrophysiological recording material.

From the measured CV values, we calculated a CSC_c_ value of 0.92 ± 0.23 mC cm^−2^ (*n* = 63), 0.64 ± 0.17 mC cm^−2^ (*n* = 7), 1.21 ± 0.29 mC cm^−2^ (*n* = 57), and 0.2038 ± 0.170 mC cm^−2^ (*n* = 13), for 20, 30, 40, and 400 *µ*m diameter electrodes, respectively. As shown in figure 5(d), the CV response of 40 *µ*m diameter ITO electrodes compared to the same size electrodes of different electrode materials used for electrical stimulation, such as platinum (Pt) and SIROF indicates the limited performance of ITO as a neural stimulation electrode material.

These *in vitro* results provide us with confidence that the performance of our ITO electrodes would be sufficiently optimal for electrophysiological recording.

#### 3.1.4. Optical transparency

The key differentiating advantage of the transparent Smart Dura from its predecessor is its enhanced optical access enabled by ITO electrodes for optical imaging directly underneath the electrode with simultaneous electrophysiological recording. To evaluate its optical performance, we measured direct light transmission using the high-resolution optical spectrometry setup illustrated in figure 6(a). A broadband light source from an incandescent light bulb was spectrally filtered with a 100 *µ*m pinhole prior to resolving the small spot size light beam to prevent stray light from coupling into the lens system. A first lens was used to collimate the otherwise divergent light beam sourced from the pinhole ahead of a second lens used to focus the light into a highly resolved light beam with 27 *µ*m beam waist. After the focusing lens, a beamsplitter was used to be able to image our sample with high resolution using a zoom lens system and a CCD camera. The focused light beam impinged onto the Smart Dura device which was carefully placed at the focal plane of the focusing lens with micromanipulator translating stages. A fiber-coupled spectrometer (200 *µ*m core diameter) was used to couple the light into the spectrometer to measure the transmission spectrum. The fiber was placed directly under the Smart Dura surface (figure 6(b)). The measurements consisted of measuring the optical transmission of the different materials of the transparent Smart Dura (ITO and PDMS + Parylene C films separately) as well as the fully integrated device at the ITO electrode locations.

**Figure 6.**
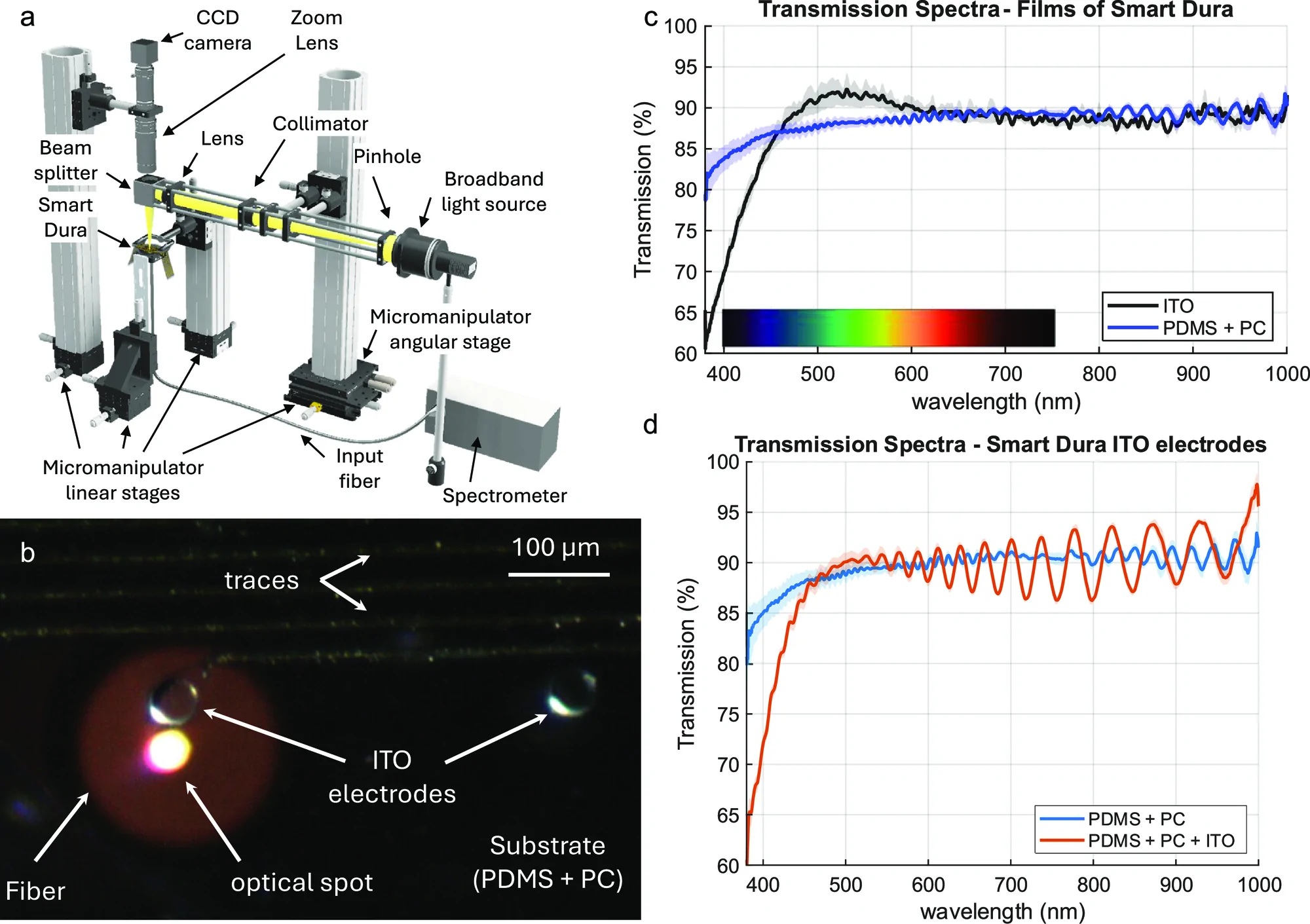
High-resolution optical spectrometry of transparent Smart Dura. (a) 3D model of our optical setup for high-resolution transmission measurements capable of providing spot sizes as low as 27 μm. (b) Micrograph illustrating the placement of our transparent Smart Dura device on top of the spectrometer-coupled fiber for transmission measurements. With micromanipulator stages we can precisely move the transparent Smart Dura to have electrode sites directly below the spot size. (c) Transmission spectrum of the transparent material films (measured separately) used for the transparent Smart Dura: ITO (black trace) for electrodes and PDMS + Parylene C (blue trace) for substrates. The sample size (*n*) of the ITO, and PDMS + PC data is *n* = 3, and *n* = 5, respectively. (d) Direct transmission spectrum of all transparent films integrated together (for both cases, *n* = 5). Note the same level of transparency as with the individual films with some Fabry–Perot resonance effects for most of the visible range. The data for the ITO film in (c) was obtained from a glass slide with 100 nm of ITO deposit on it. All the other data was obtained from a transparent Smart Dura device.

As seen in figure 6(c), the ITO films and PDMS + Parylene C (PC) films have 85%–90% optical transmission for most of the visible and near infrared (NIR) spectrum, independently. When measured together (figure 6(d)), the transmission profile follows the trend of ITO in the shorter wavelength region (<500 nm) but otherwise remains at the same level of 90% transmission, with some Fabry–Perot resonance effect [40], for the rest of the visible and NIR region. The reason why the optical transmission remains high (∼90%) instead of dropping in a cascaded fashion (10% drop from ITO and another 10% drop from PDMS + PC) is because the refractive index contrast between the ITO (n = 1.85), Parylene C (n = 1.64) and PDMS (n = 1.40) films is much lower than the refractive index contrast between ITO and air, and thus the light reflection at the interface of these films is negligible and the reflection is dominated by the index contrast at the ITO-air interface.

The high levels of transparency demonstrated here show the potential of the Smart Dura with ITO electrodes to be combined with optical stimulation or imaging techniques with simultaneous electrophysiological recording.

### 3.2. *In vivo* demonstration

Our Smart Dura with ITO electrodes, which has greater optical access than our legacy Smart Dura, was designed to further improve the performance for neural interfacing based on optical techniques while still providing simultaneous electrophysiological recording capability. To demonstrate and validate this version of the Smart Dura for these applications, we conducted *in vivo* tests involving visual stimulation experiments while recording from the primary visual cortex (V1) in an anesthetized macaque, which has a well-defined and well-studied functional topography (e.g. ocular dominance and orientation columns) that provides a reliable test-bed for electrical and imaging technologies.

#### 3.2.1. Highly transparent field of view for optical interfacing with NHP brain

In order to validate the increase in transparency demonstrated in our benchtop characterization, we performed brightfield imaging as well as OCTA of the surface of V1 in an anesthetized NHP through the Smart Dura with ITO electrodes (figure 7(a)). Figure 7(b) shows the underlying microvasculature of the entire optical window of the Smart Dura on top of the surface of V1 via OCTA. Note the reconstructed vasculature image with color-coded depth information rendered. A closer look at figure 7(b) could suggest that the green stripe-like artifacts present in the image originate from the Pt/Au metal traces. However, these are present even at locations that do not overlap with Pt/Au traces. The origin of this artifact is due to other sources such as brain motion or imperfections in the Smart Dura substrate (i.e. small scratches). This is confirmed in the side-view projections (b-scans) shown in figure S1. Figures 7(c) and (d) show a comparison between brightfield images of the entire field of view provided by the legacy Smart Dura (figure 7(c)) and the new transparent Smart Dura with ITO electrodes (figure 7(d)). Note that in figure 7(d) the ITO electrodes are not obstructing the underlying tissue. Figures 7(e) and (f) show close up images focused on the transparent ITO electrodes placed on top of the surface of the brain for 30 *µ*m (figure 7(e)) and 400 *µ*m diameter electrodes (figure 7(f)).

**Figure 7.**
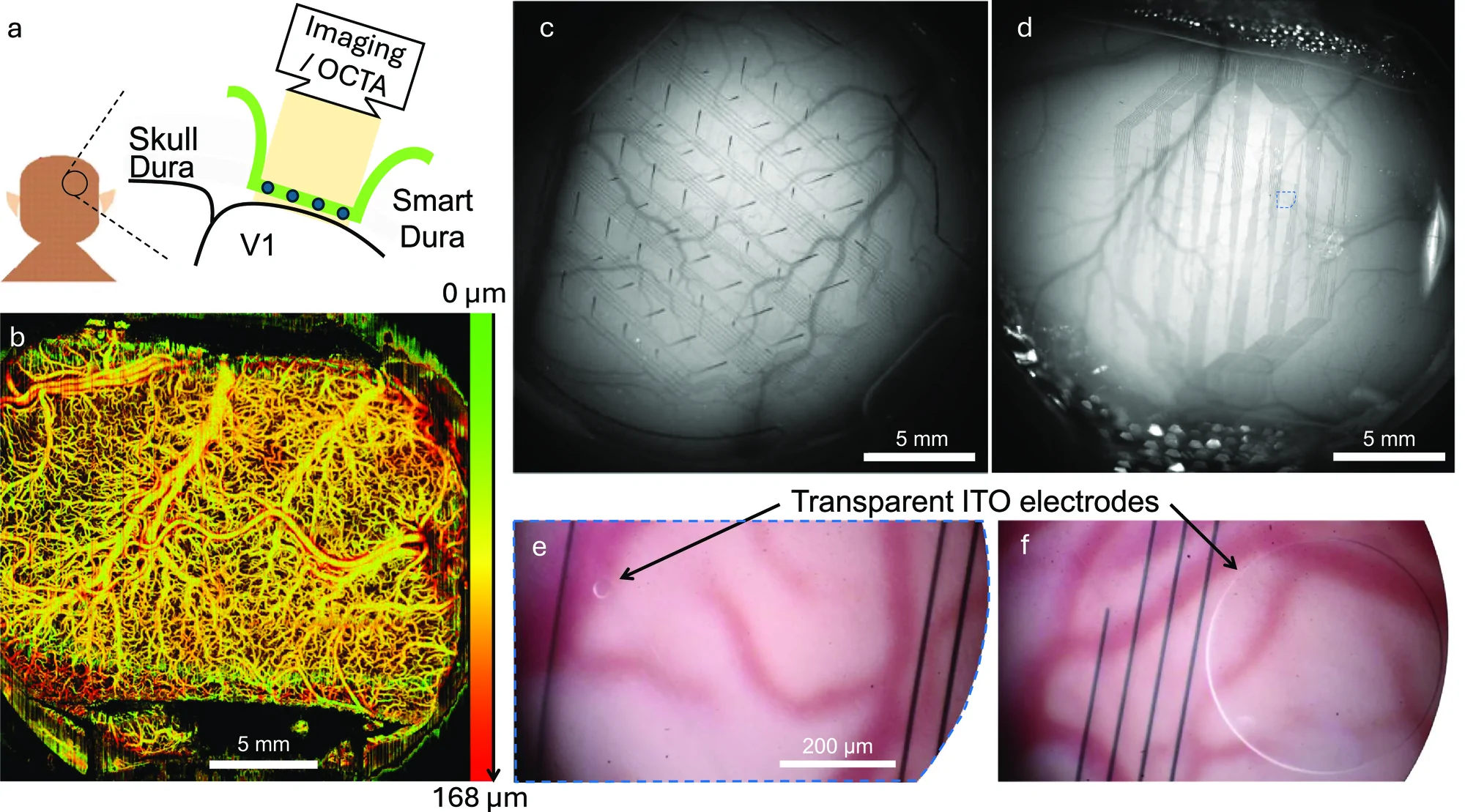
High optical access of our Smart Dura with ITO transparent electrodes for NHP brain interfacing. (a) Schematic of the implementation of transparent Smart Dura on NHP brain over a 20 mm diameter field of view. (b) Optical coherence tomography angiography (OCTA) image of the underlying microvasculature in primary visual cortex (V1) through 128-ch transparent Smart Dura with 30 *µ*m electrodes, where vessel information is depth-coded with green indicating the superficial vessels and red the deeper vessels. (c) Brightfield image of the NHP brain through legacy Smart Dura [18] presented here for direct comparison with the transparent Smart Dura with ITO electrodes. (d) Brightfield image of NHP cortex through the Smart Dura with transparent ITO electrodes. Note that the regions that are exclusively made of ITO are completely transparent. (e), (f) Brightfield image of the NHP brain through transparent Smart Dura with 30 *µ*m electrodes and 400 *µ*m ITO electrodes. Note that panels (b), (d), and (e) were obtained from the same brain region through the same transparent Smart Dura. The results in this figure qualitatively demonstrate the high optical access enabled by the highly transparent ITO electrodes and 10 *µ*m wide traces.

These brightfield and OCTA images demonstrate the high transparency of ITO electrodes *in vivo.*

#### 3.2.2. Multi-photon imaging through ITO

To quantitatively demonstrate the *in vivo* optical transparency of the ITO electrodes incorporated into our new Smart Dura device, we conducted multiphoton microscopy imaging experiments. Specifically, we performed two-photon and three-photon excitation microscopy using 920 and 1300 nm light to visualize cortical vasculature through the legacy Smart Dura and transparent Smart Dura, in an anesthetized macaque following intravenous administration of FITC-dextran (figure 8(a)). This approach enabled us to acquire high-resolution fluorescence images of microvascular structures within V1 at depths of 200 *μ*m beneath the Smart Dura devices (figures 8(c) and (e)) and up to 450 *μ*m in depth (figure S2). At the cortical surface (depth = 0 *μ*m; top panel in figure 8(c)), the image clearly delineates the position of the ITO electrodes as well as the 10 *μ*m-wide metallic traces embedded in the Smart Dura. Consistent with our previous reports [18, 19], these metallic traces cast shadows that progressively diminish with increasing depth. Importantly, in the case of the ITO electrodes, the imaging field remains completely unobstructed, allowing direct visualization and tracking of fluorescent activity immediately beneath the electrode across multiple depths. This observation is corroborated by the intensity profiles shown in figure 8(d) and the three-dimensional reconstruction of the microvasculature presented in figure 8(b). In contrast, the legacy Smart Dura design, which employs non-transparent electrodes, fails to provide optical access beneath the opaque electrode regions, as illustrated in figure 8(e) and further supported by the intensity profiles in figure 8(f). As shown in our prior publication, the shadow of a thin 10 *μ*m trace on the underlying tissue structure is negligible [19].

**Figure 8.**
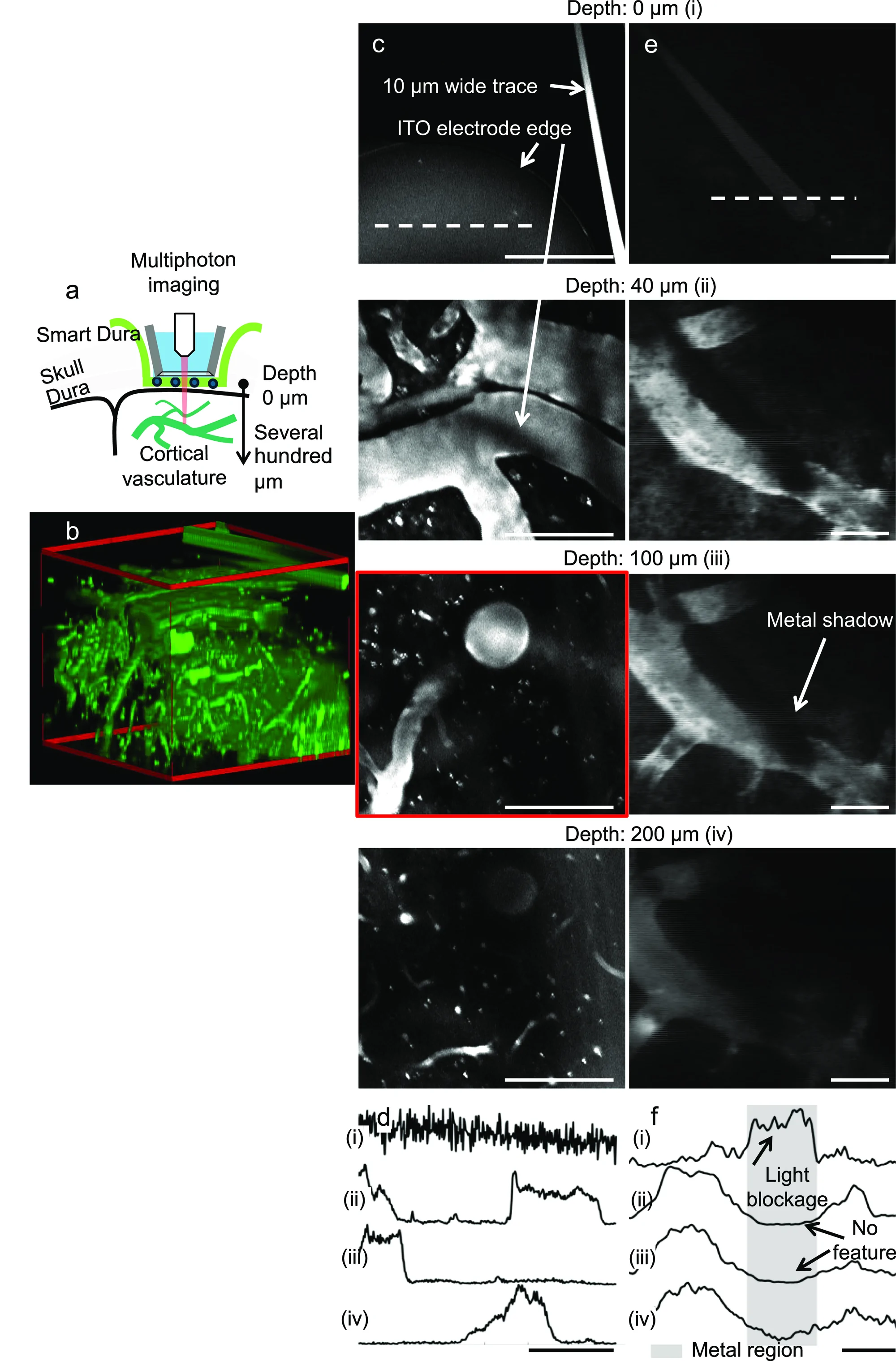
*In vivo* multi-photon microscopic imaging of vasculature within NHP brain through the Smart Dura with transparent ITO electrodes. (a) Schematic of *in vivo* multiphoton imaging of cortical vasculature through the transparent Smart Dura. (b) 3D reconstruction of the vasculature through the transparent Smart Dura substrates and ITO electrodes from a three-photon image stack, with some images shown in (c). (c) Three-photon *in vivo* fluorescent images of brain vascular through the Smart Dura with transparent ITO electrodes focused on the metal traces (depth: 0 *µ*m) and deeper in cortex (depths: 40, 100, and 200 *µ*m), showing large and micro-size vasculature directly underneath the ITO electrodes. Scale bar: 100 *µ*m. (d) Normalized greyscale intensity profiles at depths of 0, 40, 100, and 200 *µ*m (from top to bottom) along the white dash line shown in (c) at a depth of 0 *µ*m. Scale bar: 50 *µ*m. (e) Two-photon fluorescent vascular images through the legacy Smart Dura with non-transparent electrodes focused on the metal traces (depth: 0 *µ*m) and the vasculature below (depths: 40, 100, and 200 *µ*m), showing that area under the electrode is not accessible for imaging. Scale bar: 100 *µ*m. (f) Normalized greyscale intensity profiles for the images through legacy Smart Dura with non-transparent electrodes along the white dash line shown in (e) at a depth of 0 *µ*m. Scale bar: 50 *µ*m.

Analysis of these images highlights the exceptional optical transparency of the ITO electrodes, demonstrating that they introduce no additional attenuation or increase in noise compared to regions of the Smart Dura composed solely of substrate material. This high level of transparency is critical because it enables seamless integration of large-scale surface electrophysiological recordings provided by the Smart Dura with advanced multiphoton functional imaging techniques, such as calcium imaging. Calcium imaging allows visualization of single-neuron activity at depths of several hundred micrometers within cortical tissue. By combining these complementary modalities, researchers can directly investigate the relationship between local cellular dynamics occurring deep within the brain and global patterns of neural activity, thereby offering unprecedented insight into the coupling between microcircuit function and overall brain state.

#### 3.2.3. Electrophysiological recording

As described above, the dimensions of the ITO electrodes of our transparent Smart Dura were designed to be primarily sized between 20 and 40 *µ*m in diameter to provide high spatial resolution electrophysiological recording. Given the electrochemical properties of ITO and our micrometer sized electrodes, the electrochemical impedance we measured for our 30 *µ*m diameter ITO transparent Smart Dura electrodes was high (2.52 MΩ), especially compared to same size electrodes made out of Pt (495 kΩ) [37, 41], Pt-nanoparticles [42], PEDOT:PSS (15.37 kΩ) [30, 41] or SIROF (17.60 kΩ) [30, 41]. While the noise levels calculated from the EIS measurements remained low and within one order of magnitude difference to other electrode materials, the high electrochemical impedance remained concerning on whether our ITO electrodes would be able to provide reliable electrophysiological recordings of the underlying neural activity on par with our previous results from our legacy Smart Dura with Pt and PEDOT:PSS electrodes [19]. To this end, we performed the same visual stimulation experiment as we performed previously [18] to obtain a comparison with our successful previous results. As shown in figures 9(a) and (b), we conducted visual stimulation experiments in an anesthetized animal while recording neural signals from V1 using the Smart Dura with ITO electrodes. Visual stimuli consisting of chromatic and achromatic drifting square-wave gratings oriented at different angles and with a duration of 4 s were randomly interleaved and presented separately to the left and right eyes. We were able to reliably record neural activity during chromatic and achromatic visual stimulation from our 30 *µ*m diameter ITO electrodes with HG activity in the frequency range between 60–150 Hz as shown in figures 9(d) and (f). For both chromatic and achromatic stimuli, signal power increased as the stimuli were applied. We obtained consistent results with a separate transparent Smart Dura with 400 *µ*m diameter ITO electrodes, which exhibit a much lower electrochemical impedance (35.91 kΩ), as shown in figures 10(d)–(f).

**Figure 9.**
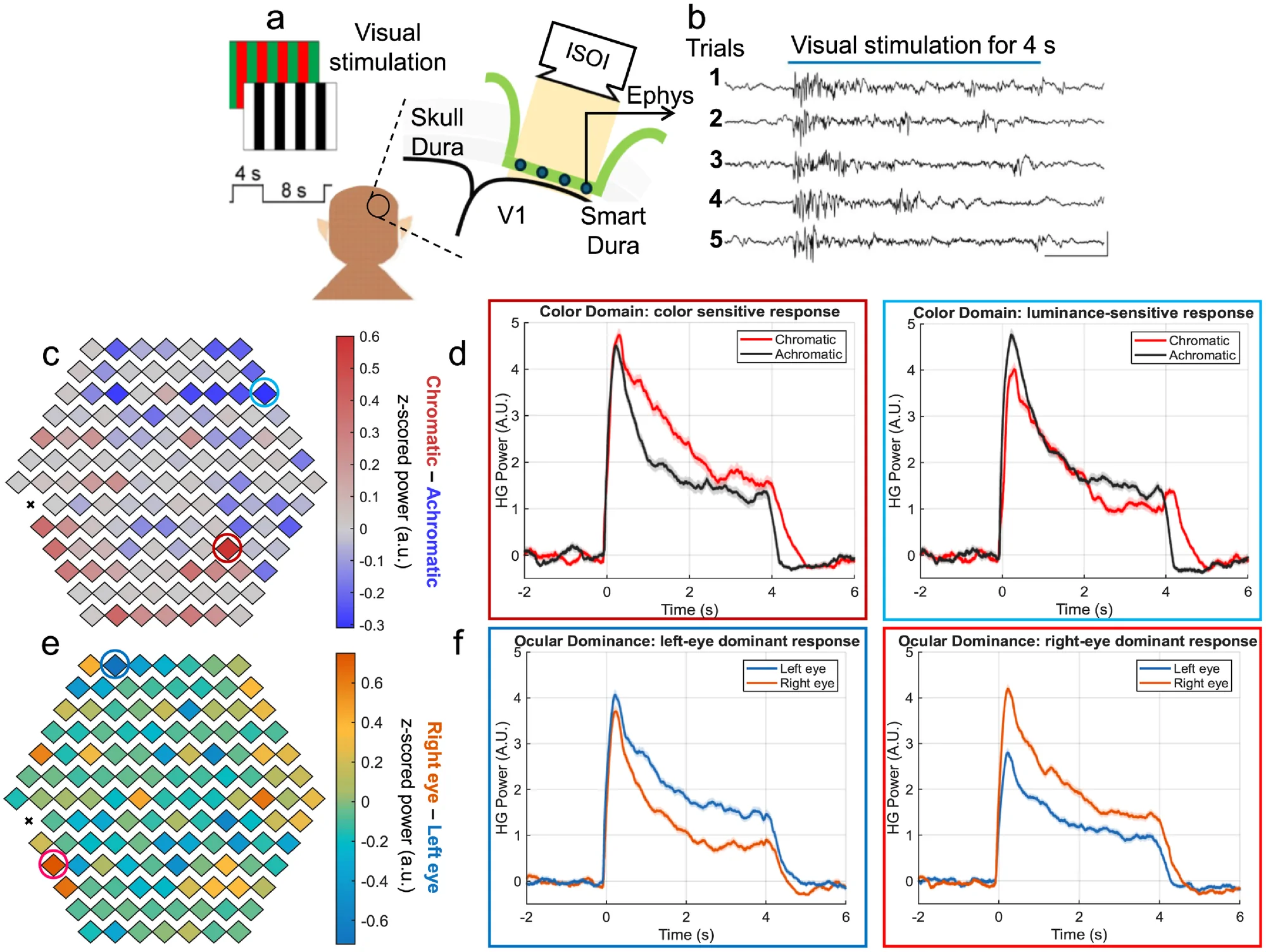
Electrophysiological recording of neural activity with our transparent Smart Dura. (a) Schematic of visual stimulation on an anesthetized animal and simultaneous wide-field intrinsic signal optical imaging (ISOI) and electrophysiological recording (Ephys) from V1. (b) Low pass filtered (*f*_c_ =250 Hz) LFP traces from a 30 *µ*m diameter ITO electrode during visual stimulation. Scale bar is 1 sec (horizontal) and 500 µV (vertical). (c) Heatmap for color domains based on electrophysiological recordings during right-eye visual stimulation. The heatmap depicts the difference in trial-averaged high-gamma (HG) (60–150 Hz) power between chromatic and achromatic stimulation (80 trials for each). Redder regions indicate strong color-sensitive responses, whereas bluer regions indicate stronger luminance-sensitive responses (d) Representative averaged HG power traces from electrodes exhibiting preferential color-sensitive responses (left red boxed panel; electrode marked with a red circle in each heatmap), and preferential luminance-sensitive responses (right blue boxed panel; electrode marked with a blue circle in each heatmap). Solid lines and shaded areas denote the mean and standard error of the mean, respectively. *n* = 80 trials for each. (e) Heatmap for ocular dominance based on electrophysiological recordings. The electrode array here is the same as in (c). The heatmap depicts the difference in trial-averaged HG power between right-eye and left-eye stimulation (160 trials for each). Redder regions indicate strong right-eye-dominant responses, whereas bluer regions indicate stronger left-eye-dominant responses. (f) Representative averaged HG power traces from electrodes, exhibiting right-eye-dominant responses (red boxed panel; electrode marked with a red circle in each heatmap), and left-eye-dominant responses (blue boxed panel; electrode marked with a blue circle in each heatmap). Solid lines and shaded areas denote the mean and standard error of the mean, respectively. *n* = 160 trials for each.

**Figure 10.**
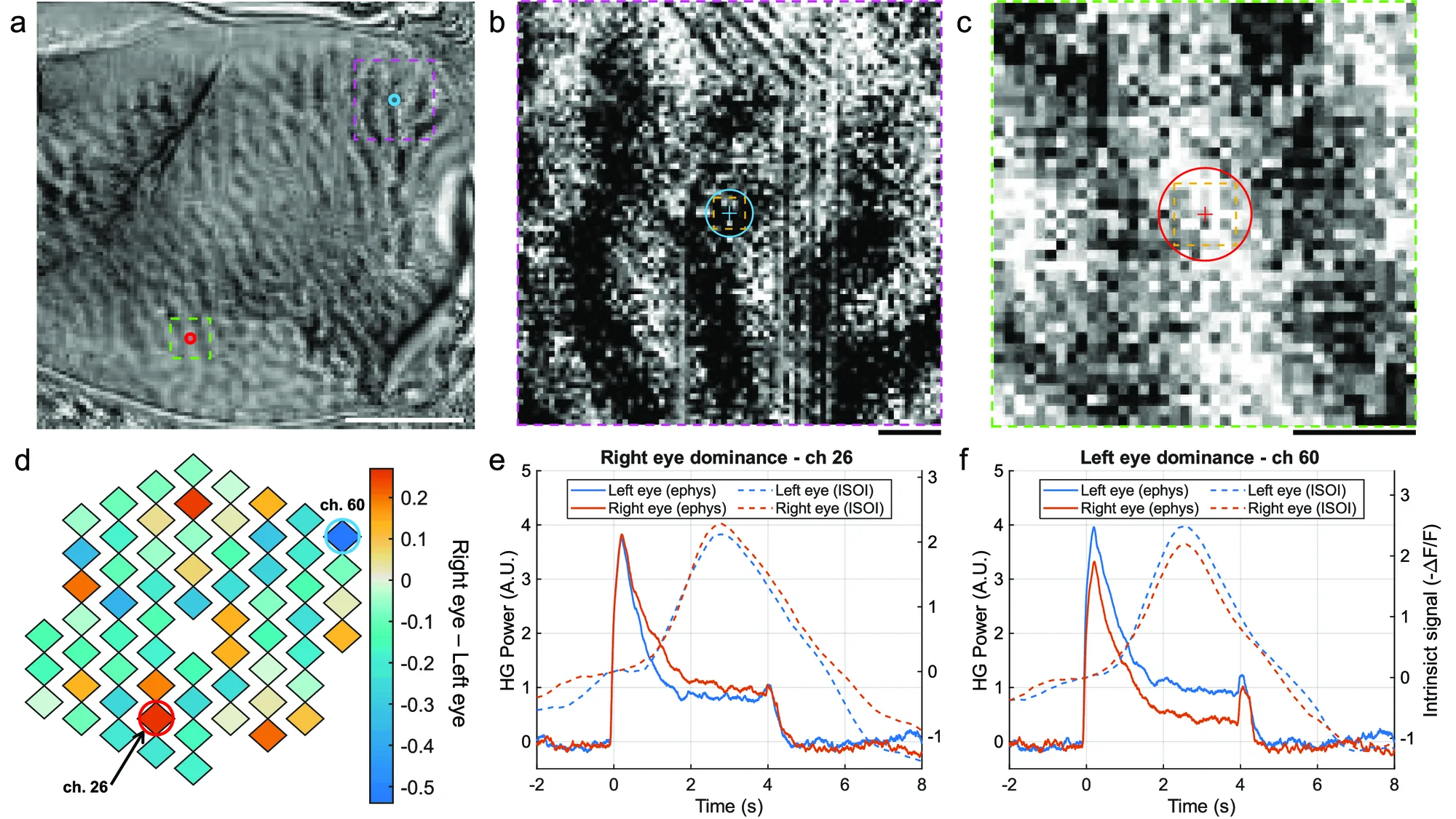
Unobstructed multimodal interfacing of primary visual cortex in NHP via wide-field ISOI and simultaneous electrophysiological through our transparent Smart Dura. (a) Ocular dominance map of V1, where dark pixels show left-eye dominant columns and light pixels show right-eye dominant columns. Scale bar: 5 mm. (b), (c) Zoomed in view of the ocular dominance map around a 400 *µ*m diameter transparent ITO electrode, whose location is indicated by the red and blue circles. Scale bar: 500 *µ*m. Note the unobstructed optical access enabled by the ITO transparent electrodes and the overlap of the electrodes with a region corresponding to a left-eye (b) and right-eye (c) dominant column, respectively. The orange dash square shows the 7 × 7 pixel grid used to average the ISOI signal in (e) and (f). (d) Heatmap for ocular dominance based on electrophysiological recordings. The heatmap depicts the difference in trial-averaged high-gamma (HG; 60–150 Hz) power between right-eye and left-eye stimulation (160 trials for each). Blue regions indicate strong left-eye dominant responses, whereas orange regions indicate stronger right-eye dominant responses. (e), (f) Trial-averaged HG power via electrophysiological and intrinsic signal via ISOI in response to right-eye and left-eye stimulation (160 trials each). (e) Shows the responses of electrodes (Ephys) and 7 × 7 grid pixels beneath the electrode (ISOI) with right-eye dominance and f) shows the responses of electrodes (Ephys) and 7 × 7 grid pixels beneath the electrode (ISOI) with left-eye dominance.

Beyond confirming the ability of the high impedance 30 *µ*m diameter and low impedance 400 *µ*m diameter ITO electrodes to capture high-gamma activity during visual stimulation, we next examined whether these recordings could support functional mapping of cortical domains. Figure 9(c) shows a heatmap of color domain organization in V1 based on trial-averaged HG power differences between chromatic and achromatic stimulation during right-eye presentation. Distinct clusters of electrodes exhibited strong color sensitivity (red regions) or luminance sensitivity (blue regions), indicating that the 30 *µ*m diameter ITO electrodes of the transparent Smart Dura can resolve fine-scale functional domains despite the higher impedance. Representative HG power traces from preferentially color-sensitive (left) and luminance-sensitive (right) electrodes are shown in figure 9(d), confirming robust and stimulus-specific responses across 80 trials per condition with a low standard error from the mean. Similarly, ocular dominance maps derived from HG power differences between right-eye and left-eye stimulation are shown in figure 9(e), revealing clear spatial segregation of eye preference consistent with known V1 organization. Figure 9(f) provides example traces from electrodes exhibiting strong right-eye or left-eye dominance, further validating the reliability of ITO-based recordings for functional mapping. Together, these results demonstrate that despite their higher electrochemical impedance, ITO microelectrodes integrated into the transparent Smart Dura can successfully capture neural dynamics at sufficient fidelity to generate high-resolution maps of color and ocular dominance domains in NHP visual cortex.

#### 3.2.4. Obstruction-free multimodal neural interfacing through ITO electrodes

To further evaluate the suitability of the transparent Smart Dura for multimodal cortical interfacing, we replicated our previously reported wide-field ISOI experiments paired with simultaneous large-scale electrophysiological recordings in V1 [18]. As illustrated in figure 9(a), intrinsic hemodynamic responses to visual stimulation, that show neurovascular coupling effects, were imaged through the Smart Dura while neural signals were simultaneously recorded using the transparent Smart Dura with 400 *µ*m diameter ITO electrodes. The high optical transparency of the ITO contacts enabled unobstructed visualization of functional cortical activity, allowing us to resolve ocular dominance columns directly beneath the electrodes without the shadows or blind spots introduced by conventional opaque electrode materials.

As shown in figures 10(a)–(c), we generated clear ocular dominance maps via ISOI that remained continuous across electrode boundaries. However, in contrast to figure 7(b), the stripe-like artifacts in figures 10(a) and (b) do originate from the Pt/Au traces and the shadow they cast onto the surface of the brain coupled with brain micromotion. Despite the presence of this artifact, the ocular dominance columns extracted (the optical targets for this study) have a width of 400–500 *µ*m, much larger than the size of the Pt/Au stripe shadow and thus having no practical impact on the effective optical access of the transparent Smart Dura. Corresponding ocular dominance maps computed from trial-averaged HG power (60–150 Hz) revealed spatial patterns that closely matched the ISOI-derived maps (figure 10(d)). A direct comparison of individual electrodes and the underlying cortical pixels highlighted a one-to-one correspondence: electrodes positioned over right-eye–dominant regions (red circle in figure 10(c)) exhibited HG responses with greater amplitude during right-eye stimulation, whereas electrodes over left-eye–dominant regions (blue circle in figure 10(b)) showed the opposite preference. These findings were further confirmed by comparing the electrophysiological traces from each electrode with the average intrinsic signal from the pixels directly beneath the electrode (orange dash squares in figures 10(b) and (c)), demonstrating strong consistency across the two modes of neural interfacing (figures 10(e) and (f)).

In terms of photoelectric artifacts arising from the use of light sources (e.g. LEDs, lasers, etc) in combination with electrophysiological recording, our mitigation strategy consisted of using the light source in a continuous wave operation, instead of being pulsed. In these experiments, we turned on the light source (i.e. the LED) before the onset of electrophysiological recording and it was kept on throughout the experiment. Thus, the photoelectric artifact was present both in the baseline recording and also after the onset of visual stimulation during the experiment. Therefore, as it can be seen from figures 9(b)–(f) and 10(e) and (f) the artifact is averaged out and does not directly affect our electrophysiological recordings.

Together, the findings in figures 10 and S3 highlight that transparent ITO electrodes not only preserve high-quality electrophysiological recordings but also enable truly obstruction-free optical imaging across the device footprint. The resulting hybrid maps derived from ISOI and HG activity strongly align across modalities, underscoring the capability of the transparent Smart Dura to support large-scale, high-fidelity, multimodal neural interfacing in NHP cortex.

## 4. Discussion

In this work we introduced the transparent Smart Dura, a customizable, microfabricated multimodal neural interface that builds upon the legacy Smart Dura platform by combining large-scale, high-resolution electrophysiology with enhanced optical access enabled through ITO-based transparent electrodes. Our scalable fabrication and packaging approach supports high-throughput production with flexible and versatile electrode configurations, tailored for specific neuroscience applications.

### 4.1. Complete effective optical access and unmatched scalability

Following the logic presented in our previous publication [19], our 1st generation Smart Dura has an effective optical access superior to the apparent optical access enabled by microfabricated 10 *µ*m wide traces that minimally block light and enable light to pass around them. However, the larger features (tapers and electrodes) made of opaque metal block light within the field of view and prevent the Smart Dura from providing complete optical access. By replacing these metal features with ITO transparent films, we improve theoptical access.

As shown in the optical transparency section (figure 6), the transmission of light through the ITO electrodes does not decrease nor is it negatively affected compared to that of the Smart Dura substrates (PDMS and Parylene C). Therefore, in the context of the optical access metric we articulated in our previous publication [19], the ITO electrodes do not obstruct light. Therefore, as shown in figure S4, our transparent Smart Dura devices with opaque metal traces ⩽10 *µ*m in width and electrodes and routing tapers made of ITO exhibit 100% effective optical access, based on our definition of effective optical access (equation (6)). In other words, our transparent Smart Dura provides an effectively unobstructed optical field of view to the surface of the NHP brain for optical recording and perturbation with simultaneous high density and high resolution electrophysiological recording capabilities.

More importantly, this has beneficial implications in terms of the scalability in the number and spatial density of the transparent Smart Dura. As discussed in our previous publication [19], the extremely narrow (10 *µ*m) Pt/Au traces produced by our lithography-based fabrication process further enhance scalability by minimizing the footprint of conductive routing. While 10 *µ*m wide traces were conservatively chosen in our design to maximize the microfabrication yield, the traces can be easily miniaturized to 1 *µ*m wide enabled by our high-resolution photolithography process. As shown in figures S4(g) and (h), when the array density is increased, the simulated traditional opaque-electrode designs rapidly occlude large fractions of the cortical surface, fragmenting the optical field of view and undermining the ability to interpret patterns such as ocular dominance or orientation domains. In contrast, arrays composed of transparent ITO electrodes and thin traces retain nearly unobstructed visibility across the entire field, even at high channel counts. Quantifying these scenarios reveals an optical access of 82.28% for the opaque electrode versus a 100% optical access for the transparent case over the same 1.69 × 1.69 mm^2^ field of view. This combination of transparent electrodes and ultra-thin trace geometry therefore provides a practical path to dense, wide coverage microelectrode arrays that remain fully compatible with ISOI and other optical modalities, offering unmatched scalability for future multimodal neural interfacing applications.

### 4.2. Reliable electrophysiology recording despite high electrode impedance values

As discussed earlier, electrodes with an impedance magnitude of less than 1 MΩ at 1 kHz are typically used to record extracellular electrophysiological signals, primarily to achieve high signal-to-noise ratio, given the typical input impedance of existing electrical neural amplifiers [28]. Increasing the size of ITO electrodes can reduce impedance below this threshold, as demonstrated by 400 *µ*m diameter electrodes exhibiting |*Z*| = 35.91 ± 1.68 kΩ at 1 kHz, but this comes at the cost of reduced spatial resolution. However, our noise characterization results reveal a low noise floor of < 30 *µ*V_rms_ for 40 *µ*m diameter ITO electrodes with an impedance magnitude of 1.6 MΩ. Notably, despite the drastic difference in impedance magnitude between the smallest and largest electrodes, the thermal noise of the smallest electrode (i.e. 29.87 *µ*V_rms_) is still well below typical electrophysiological signal amplitudes. These findings indicate that our micro-size ITO electrodes (20–40 *µ*m in diameter) maintain a low noise floor.

Importantly, the *in vivo* experiments confirm that this theoretical expectation translates into practical performance. HG activity (60–150 Hz) was reliably recorded during visual stimulation, and functional maps of color domains and ocular dominance were successfully generated (figure 9). Additionally, these results are comparable with those using lower impedance 20 *µ*m diameter PEDOT:PSS electrodes from our previous work [18]. The ability to resolve fine-scale cortical organization despite elevated impedance values demonstrates that electrode impedance alone is not a limiting factor when the noise floor remains low and signal amplitudes are sufficiently large. This suggests that transparent ITO electrodes can achieve robust electrophysiology without compromising functional mapping capabilities, even at micrometer-scale dimensions.

### 4.3. Unmatched scalability without compromising optical access enabled by ITO electrodes

In addition to providing localized optical access directly beneath the electrodes, a key advantage of the transparent Smart Dura lies in its ability to scale electrode count and density without sacrificing optical access, a limitation that has constrained multimodal implants employing opaque metals such as Pt or Au. Because the ITO electrodes and 10 *µ*m wide traces are effectively transparent, wide-field or multi-photon imaging can be performed seamlessly through the device footprint, even when electrode density is increased. In the context of studying primary visual cortex, figure S5 illustrates this fundamental benefit: when an ITO electrode overlays an ocular dominance column, ISOI signals from the pixels directly beneath the electrode remain fully accessible (figures S5(a) and (b)), preserving an accurate ocular dominance readout. In contrast, if we simulate the effect of an opaque electrode by covering the same region on the obtained image in our post-experiment image processing, we observe a complete loss of the underlying ISOI information (figures S5(c) and (d)), and this lost information cannot be recovered from the neighboring pixels. This demonstrates that electrode opacity introduces irreversible blind spots in functional imaging, fundamentally limiting scalability for multimodal cortical interfacing. It should be noted that when the electrodes are small (e.g. 50 *µ*m metal diameter), as shown in our previous work, the occluded regions may seem to be negligible when the electrode pitch is large (e.g. 1.8 mm) [19]. The sparse placement of such small electrodes may not significantly compromise the overall optical access [31, 43]. However, this becomes a serious issue when scaling up the number and density of electrodes to achieve much desired higher resolution electrophysiological recording. Our transparent ITO electrode design addresses this issue by providing optical access through the electrodes, while still enabling unmatched scalability.

## 5. Conclusion

Building on our previously established Smart Dura platform, we demonstrate here that incorporating transparent ITO electrodes substantially expands its optical functionality. By replacing opaque metal electrodes and wide interconnect regions with highly transparent, conductive ITO, the new transparent Smart Dura achieves effectively 100% optical access, enabling localized optical imaging and modulation (i.e. via optogenetics) directly beneath individual electrodes without sacrificing electrical performance. As the design scales to higher electrode counts, this advancement becomes increasingly impactful: the number and density of electrodes can be increased without compromising optical access, thereby supporting fully high‐resolution, multimodal mapping of large cortical areas. Together, these advancements highlight the transparent Smart Dura as a uniquely capable neural interface for next‐generation, high‐density, multimodal neural interfacing of brain function in NHPs, with clear translational relevance for human neuroscience and neuromodulation technologies.

## Data Availability

The data that support the findings of this study will be openly available following an embargo at the following URL/DOI: https://doi.org/10.1184/R1/33040301. Supplemental Information available at: https://doi.org/10.1088/1741-2552/ae8eb3/data1.
